# Implementation strategies to improve statin utilization in individuals with hypercholesterolemia: a systematic review and meta-analysis

**DOI:** 10.1186/s13012-021-01108-0

**Published:** 2021-04-13

**Authors:** Laney K. Jones, Stephanie Tilberry, Christina Gregor, Lauren H. Yaeger, Yirui Hu, Amy C. Sturm, Terry L. Seaton, Thomas J. Waltz, Alanna K. Rahm, Anne Goldberg, Ross C. Brownson, Samuel S. Gidding, Marc S. Williams, Michael R. Gionfriddo

**Affiliations:** 1 Genomic Medicine Institute, Geisinger, 100 N Academy Ave., Danville, PA 17822 USA; 2 Center for Pharmacy Innovation and Outcomes, Geisinger, Danville, PA USA; 3 Bernard Becker Medical Library, Washington University in St. Louis, St. Louis, MO USA; 4 Population Health Sciences, Geisinger, Danville, PA USA; 5 University of Health Sciences and Pharmacy in St. Louis, St. Louis, MO USA; 6 Population Health, Mercy Clinic—East Communities, St. Louis, MO USA; 7 Eastern Michigan University, Ypsilanti, MI USA; 8 Washington University School of Medicine, Washington University in St. Louis, St. Louis, MO USA; 9 Prevention Research Center in St. Louis, Brown School, Washington University in St. Louis, St. Louis, MO USA; 10 Department of Surgery (Division of Public Health Sciences) and Alvin J. Siteman Cancer Center, Washington University School of Medicine, Washington University in St. Louis, St. Louis, MO USA

**Keywords:** Statin, Hypercholesterolemia, Implementation strategies, Uptake, Meta-analysis

## Abstract

**Background:**

Numerous implementation strategies to improve utilization of statins in patients with hypercholesterolemia have been utilized, with varying degrees of success. The aim of this systematic review is to determine the state of evidence of implementation strategies on the uptake of statins.

**Methods and results:**

This systematic review identified and categorized implementation strategies, according to the Expert Recommendations for Implementing Change (ERIC) compilation, used in studies to improve statin use. We searched Ovid MEDLINE, Embase, Scopus, Cochrane Database of Systematic Reviews, Cochrane Central Register of Controlled Trials, and Clinicaltrials.gov from inception to October 2018. All included studies were reported in English and had at least one strategy to promote statin uptake that could be categorized using the ERIC compilation. Data extraction was completed independently, in duplicate, and disagreements were resolved by consensus. We extracted LDL-C (concentration and target achievement), statin prescribing, and statin adherence (percentage and target achievement). A total of 258 strategies were used across 86 trials. The median number of strategies used was 3 (SD 2.2, range 1–13). Implementation strategy descriptions often did not include key defining characteristics: temporality was reported in 59%, dose in 52%, affected outcome in 9%, and justification in 6%. Thirty-one trials reported at least 1 of the 3 outcomes of interest: significantly reduced LDL-C (standardized mean difference [SMD] − 0.17, 95% CI − 0.27 to − 0.07, *p* = 0.0006; odds ratio [OR] 1.33, 95% CI 1.13 to 1.58, *p* = 0.0008), increased rates of statin prescribing (OR 2.21, 95% CI 1.60 to 3.06, *p* < 0.0001), and improved statin adherence (SMD 0.13, 95% CI 0.06 to 0.19; *p* = 0.0002; OR 1.30, 95% CI 1.04 to 1.63, *p* = 0.023). The number of implementation strategies used per study positively influenced the efficacy outcomes.

**Conclusion:**

Although studies demonstrated improved statin prescribing, statin adherence, and reduced LDL-C, no single strategy or group of strategies consistently improved outcomes.

**Trial registration:**

PROSPERO CRD42018114952.

**Supplementary Information:**

The online version contains supplementary material available at 10.1186/s13012-021-01108-0.

Contributions to the literature

- A variety of implementation strategies have been used to promote statin uptake.
- Lack of generalizability of implementation strategies to improve statin use is due in part to lack of detailed reporting of these strategies in the literature.
- No single implementation strategy appears to be associated with improved outcomes when compared with others.
- Multiple implementation strategies are likely to be required to improve statin utilization.

## Introduction

Statin medications reduce low-density lipoprotein cholesterol (LDL-C) blood concentrations and cardiovascular events in patients with hypercholesterolemia, and guidelines recommend statin therapy to lower LDL-C in patients who are at risk for developing or have known atherosclerotic cardiovascular disease [1]. Despite evidence for the benefits of statins, the medications are widely underutilized [2–6]. Previous studies highlight both patient- and prescriber-barriers to statin use including side effects, competing medical conditions, busy clinics, and patient reluctance affecting adherence to prescribed medications [7–9]. Lack of adherence is associated with increased mortality in a dose dependent relationship [10].

Implementation strategies can be used to promote the uptake of interventions, such as statin therapy, and are defined as “methods or techniques used to enhance the adoption, implementation, and sustainability of a clinical program or practice” [11]. Numerous implementation strategies have been attempted to improve utilization of statins, all with varying degrees of success. These studies have targeted a variety of actors (e.g., patients, clinicians, or systems) and employed a variety of implementation strategies (e.g., education, reminders, or financial incentives). A computer-based clinical decision support system to aid in prescribing of evidence-based treatment for hyperlipidemia, which targeted clinicians, was found to significantly reduce blood LDL-C concentrations [12]. However, when providing financial incentives to providers, patients, or both, a study found that only the combination incentive was successful in reducing LDL-C levels to target [13]. The absolute and comparative effectiveness of these strategies, however, is unclear. Knowing which strategies are most effective can facilitate the uptake of statins and lead to reduce mortality.

To address this issue, we aimed to address the following key questions:

1. What implementation strategies have been used to promote the uptake of statins?
2. How completely are the implementation strategies utilized reported in studies designed to promote statin uptake?
3. Which implementation strategy, or combination of strategies, is (are) the most effective at promoting the uptake of statins?

We conducted a systematic review of studies aimed at improving statin use and categorized implementation strategies by the Expert Recommendations for Implementing Change (ERIC) compilation [14]. Our primary objective was to better understand the impact of specific implementation strategies on the utilization of statins in patients with hypercholesterolemia. Our secondary objective was to evaluate statin adherence, statin prescribing, and lowering of LDL-C after intervention.

## Methods

This registered (PROSPERO CRD42018114952) systematic review adhered to the reporting guidelines of the Preferred Reporting Items for Systematic Reviews and Meta-analyses (PRISMA) statement [15].

### Search strategy

A medical librarian (L.H.Y.) searched the literature for records including the concepts of hypercholesterolemia, hyperlipidemia, and statins. The search strategies used a combination of keywords and controlled vocabulary and searched the following databases from inception to October 2018: MEDLINE, Embase, Scopus, Cochrane Database of Systematic Reviews, Cochrane Central Register of Controlled Trials, and Clinicaltrials.gov. References were imported into Endnote™ and duplicates were identified and removed. An example of the search string can be found in Table 1 and the fully reproducible search strategies for each database can be found in Additional file 1: Appendix 1.

**Table 1:** Example search string

Database	Search string
Embase	('hypercholesterolemia'/exp OR 'familial hypercholesterolemia'/exp OR hypercholesterolemia:ti,ab,kw OR cholesteremia:ti,ab,kw OR cholesterinemia:ti,ab,kw OR cholesterolemia:ti,ab,kw OR hypercholesteremia:ti,ab,kw OR hypercholesterinaemia:ti,ab,kw OR hypercholesterinemia:ti,ab,kw OR hypercholesterolaemia:ti,ab,kw OR (('high cholesterol' NEAR/1 level*):ti,ab,kw) OR ((elevated NEAR/1 cholesterol*):ti,ab,kw) OR 'hyperlipidemia'/exp OR 'familial hyperlipemia'/exp OR hyperlipemia*:ti,ab,kw OR hyperlipaemia:ti,ab,kw OR hyperlipemia:ti,ab,kw OR hyperlipidaemia:ti,ab,kw OR hyperlipidaemias:ti,ab,kw OR hyperlipidemias:ti,ab,kw OR hyperlipidemic:ti,ab,kw OR lipaemia:ti,ab,kw OR lipemia:ti,ab,kw OR lipidaemia:ti,ab,kw OR lipidemia:ti,ab,kw) AND ('hydroxymethylglutaryl coenzyme a reductase inhibitor'/exp OR 'hydroxymethylglutaryl coenzyme a reductase inhibitor':ti,ab,kw OR 'hydroxymethylglutaryl-coa inhibitors':ti,ab,kw OR 'hydroxymethylglutaryl-coenzyme a inhibitors':ti,ab,kw OR 'hmg coa reductase inhibitor':ti,ab,kw OR 'hmg coenzyme a reductase inhibitor':ti,ab,kw OR 'hmg coa reductase inhibitors':ti,ab,kw OR 'hydroxymethylglutaryl coa reductase inhibitors':ti,ab,kw OR 'hydroxymethylglutaryl-coa reductase inhibitors':ti,ab,kw OR statin:ti,ab,kw OR statins:ti,ab,kw OR vastatin:ti,ab,kw)

### Study selection

We included studies reported in English, regardless of the country where the study was conducted, that had at least one strategy promoting statin uptake that could be categorized using the ERIC compilation [14, 16]. Seven manuscripts were excluded for this reason. The ERIC compilation was created so that researchers have a standardized way to name, define, and categorize implementation strategies. The ERIC compilation was selected for use in this review because the implementations strategies in the included articles most closely matched the ERIC taxonomy compared to other available choices [17]. For key questions 1 and 2, we did not limit inclusion based on study design or outcome. For key question 3, we limited inclusion to randomized controlled trials (RCTs). Studies were excluded for key questions if full text was not available.

Search results were uploaded into systematic review software (DistillerSR, Ottawa, Canada). In the first round of screening, abstracts and titles were evaluated for inclusion. Following abstract screening, eligibility was assessed through full-text screening. Prior to both abstract and full text screening, reviewers underwent training to ensure a basic understanding of the background of the field and purpose of the review as well as comprehension of the inclusion and exclusion criteria. The initial 20 abstracts were reviewed independently and then discussed as a group. Eligibility at both levels (abstract and full-text) was assessed independently and in duplicate (L.K.J., S.T., L.R.F., and C.G.). Disagreements at the level of abstract and full text screening were resolved by consensus. If consensus could not be achieved between the two reviewers, a third reviewer arbitrated (M.R.G., T.W., or T.S.).

### Data collection

The following characteristics were extracted from included studies: first author, year of publication, location, age of patient population (adult vs. child), study design, implementation strategies, inclusion and exclusion criteria, and any of the following outcomes: statin prescribing or use, statin adherence, or LDL-C measurements.

#### Key question 1: what implementation strategies have been used to promote the uptake of statins?

We first summarized and described the populations, interventions, comparisons, and outcomes presented for all studies that reported at least one implementation strategy that could be mapped to the ERIC compilation. The ERIC compilation of nine implementation strategies categories (73 total strategies) was applied to each of the interventions to (1) count the total number of strategies and (2) describe how complete each implemented strategy was defined. One study team member, who was an author on the original ERIC compilation, ensured validity of the categories selected (T.W.) [14].

#### Key question 2: how completely are the implementation strategies utilized reported in studies designed to promote statin uptake?

Based on guidance from proctor and colleagues, we assessed the degree to which each strategy was completely reported including actor, action, action target, temporality, dose, implementation outcome affected, and justification (Table 2) [11].

**Table 2:** Summary of the implementation strategies’ defining characteristics

Characteristics	Definition	% (N)
Actor	Identify who enacts the strategy	98% (254/258)
Action	Specific actions, steps, or processes that need to be enacted	100% (258/258)
Action Target	1) Specify targets according to conceptual models of implementation 2) Identify unit of analysis for measuring implementation outcomes	95% (245/258)
Temporality	Specify when the strategy is used	59% (151/258)
Dose	Specify dosage of implementation strategy	52% (134/258)
Implementation outcome affected	Identify and measure the implementation outcome(s) that are affected by each strategy	9% (23/258)
Justification	Justification for choice of implementation strategies	6% (16/258)

Characteristics and definitions were utilized from Proctor 2013. The justification definition was adjusted to reflect an argument for the implementation strategy by noting an implementation science framework or guidance and not an evidence-base for the intervention

#### Key question 3: which implementation strategy, or combination of strategies, is (are) the most effective at promoting the uptake of statins?

When present, we extracted data related to statin prescribing, statin adherence, and LDL-C reported from included RCTs. All outcomes were collected at intervention completion. Statin prescribing or use included all orders for statin medications. Statin adherence included only objective measures of adherence by either medication possession ratio (MPR) or proportion of days covered (PDC) [18]. MPR or PDC were captured as a percentage or attainment of greater than 80% adherence. LDL-C levels were recorded as LDL-C measured or achievement of an LDL-C target.

### Risk of bias assessment

The Cochrane Collaboration’s risk of bias tool version 2 to evaluate methodological quality of studies included in the meta-analysis for key question 3 [19]. The risk of bias in included studies was assessed in duplicate by two reviewers (L.K.J. and L.R.F.) working independently. Any disagreements were resolved by consensus; if consensus was unable to be achieved, a third reviewer arbitrated (M.R.G.).

### Statistical analysis

Standardized mean differences (SMDs) with corresponding 95% CIs were estimated for continuous outcomes, and odds ratios (ORs) and 95% confidence intervals (CIs) were calculated for binary outcomes from included studies. Publication bias was evaluated by Egger’s test [20]. Variability between included studies was assessed by heterogeneity tests using *I*^2^ statistic [21]. If overall results showed significant heterogeneity, potential sources of heterogeneity were explored by subgroup analysis. All analyses were conducted using RStudio (Version 1.0.136) using the “Meta” and “Metafor” package.

## Results

### Description of study selection

**Fig. 1:**
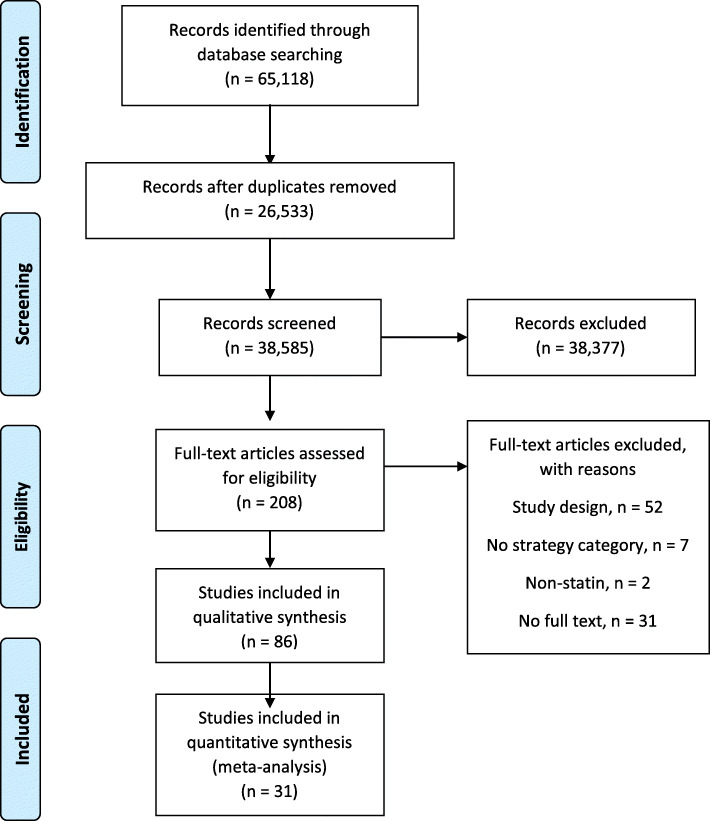
PRISMA flow diagram

We initially identified 65,118 studies. After removing duplicates, we identified 38,585 unique citations (Fig. 1). Through abstract and title screening, 208 reports were identified for full-text review. During full-text review, 86 were selected for inclusion [12, 13, 22–105]. A complete list of excluded full-text studies with rationale for exclusion is available in Additional file 1: Appendix 2.

### Description of studies

Table 3 describes the included studies (more details are included in Additional file 1: Appendix 3). Almost all the implementation strategies targeted adults (two studies included pediatric patients), half were implemented in the USA, and almost all were conducted in individuals with hypercholesterolemia (two studies were conducted in individuals with familial hypercholesterolemia).

**Table 3:** Study demographics

Year	Author last name	Location	Population	Study design	Outcomes measured	Included in meta-analysis
1996	Schectman [34]	United States	Adult	RCT	LDL-C, Statin Adherence	✓
1997	Bogden [65]	United States	Adult	RCT	LDL-C	✓
2000	Nordmann [37]	Switzerland	Adult	RCT	Statin Prescribing	✓
2000	Nguyen [38]	France	Adult	RCT	LDL-C	✓
2000	Faulkner [71]	United States	Adult and Child	RCT	LDL-C	✓
2005	Rachmani [36]	Israel	Adult	RCT	LDL-C, Statin Prescribing	✓
2006	Lester [41]	United States	Adult	RCT	LDL-C	✓
2006	Lee [78]	United States	Adult	RCT	LDL-C	✓
2007	Khanal [77]	United States	Adult	RCT	LDL-C, Statin Prescribing	✓
2008	Riesen [35]	Switzerland	Adult	RCT	LDL-C	✓
2009	Stacy [31]	United States	Adult	RCT	Statin Adherence	✓
2009	Willich [89]	Germany	Adult	RCT	LDL-C	✓
2009	McAlister [101]	Canada	Adult	RCT	LDL-C, Statin Prescribing	✓
2010	Webster [23]	Australia	Adult	RCT	LDL-C	✓
2010	Villeneuve [103]	Canada	Adult	RCT	LDL-C, Statin Prescribing	✓
2012	Nieuwkerk [86]	Netherlands	Adult	RCT	LDL-C	✓
2013	Zamora [12]	Spain	Adult	RCT	LDL-C	✓
2013	Kooy [42]	Netherlands	Adult	RCT	Statin Adherence	✓
2013	Kardas [43]	Poland	Adult	RCT	Statin Adherence	✓
2013	Goswami [72]	United States	Adult	RCT	Statin Adherence	✓
2014	McAlister [83]	Canada	Adult	RCT	LDL-C, Statin Prescribing	✓
2014	Lowrie [100]	United Kingdom	Adult	RCT	Statin Prescribing	✓
2015	Mols [54]	Denmark	Adult	RCT	LDL-C	✓
2015	Asch [13]	United States	Adult	RCT	LDL-C	✓
2015	Patel [82]	Australia	Adult	RCT	LDL-C	✓
2016	Jakobsson [44]	Sweden	Adult	RCT	LDL-C, Statin Prescribing	✓
2016	Damush [79]	United States	Adult	RCT	Statin Adherence	✓
2018	Choudhry [76]	United States	Adult	RCT	LDL-C, Statin Adherence	✓
2018	Mehrpooya [80]	Iran	Adult	RCT	LDL-C	✓
2018	Martinez [81]	Spain	Adult	RCT	LDL-C	✓
2018	Osborn [104]	United Kingdom	Adult	RCT	LDL-C, Statin Prescribing	✓
1996	Lindholm [39]	Sweden	Adult	RCT	LDL-C	
2003	Sebregts [99]	Netherlands	Adult and Child	RCT	LDL-C	
2007	Choe [95]	United States	Adult	RCT	LDL-C, Statin Adherence	
2008	Hung [90]	Taiwan	Adult	RCT	LDL-C, Statin Prescribing	
2010	Bhattacharyya [62]	Canada	Adult	RCT	LDL-C, Statin Prescribing	
2013	Dresser [55]	Canada	Adult	RCT	LDL-C	
2013	Brath [60]	Austria	Adult	RCT	LDL-C, Statin Adherence	
2013	Derose [85]	United States	Adult	RCT	Statin Adherence	
2005	Straka [28]	United States	Adult	Nonrandomized Clinical Trial	LDL-C	
2005	Paulos [105]	Chile	Adult	RCT	LDL-C, Statin Adherence	
2006	Vrijens [24]	Belgium	Adult	RCT	Statin Adherence	
2015	Persell [102]	United States	Adult	RCT	LDL-C, Statin Prescribing	
2017	Bosworth [61]	United States	Adult	RCT	LDL-C, Statin Adherence	
2018	Etxeberria [53]	Spain	Adult and Child	RCT	Statin Prescribing	
1995	Shaffer [94]	United States	Adult	Observational	LDL-C	
1997	Shibley [32]	United States	Adult	Observational	LDL-C	
1999	Schwed [33]	Switzerland	Adult	Observational	LDL-C, Statin Adherence	
2000	Robinson [92]	United States	Adult	Observational	LDL-C, Statin Prescribing	
2000	Birtcher [93]	United States	Adult	Observational	Statin Prescribing	
2001	Ford [52]	United Kingdom	Adult	Observational	Statin Prescribing	
2002	Viola [25]	United States	Adult	Observational	LDL-C, Statin Prescribing	
2002	Geber [50]	United States	Adult	Observational	LDL-C	
2002	Gavish [51]	Israel	Adult	Observational	LDL-C, Statin Adherence	
2002	Hilleman [70]	United States	Adult	Observational	LDL-C; Statin Prescribing	
2003	Truppo [27]	United States	Adult	Observational	LDL-C; Statin Adherence	
2003	Ryan [98]	United States	Adult	Observational	LDL-C; Statin Prescribing	
2004	Hilleman [45]	United States	Adult and Child	Observational	LDL-C	
2004	de Velasco [56]	Spain	Adult	Observational	LDL-C, Statin Prescribing	
2004	Lappé [69]	United States	Adult	Observational	Statin Prescribing	
2005	Harats [47]	Israel	Adult	Observational	LDL-C	
2005	Bassa [63]	Spain	Adult	Observational	LDL-C	
2005	Brady [91]	United Kingdom	Adult	Observational	Statin Prescribing	
2005	McLeod [96]	United Kingdom	Adult	Observational	Statin Adherence	
2005	Rabinowitz [97]	Israel	Adult	Observational	LDL-C	
2006	de Lusignan [57]	United Kingdom	Adult and Child	Observational	Statin Prescribing	
2006	Rehring [66]	United States	Adult	Observational	LDL-C	
2007	Goldberg [48]	United States	Adult	Observational	LDL-C	
2008	Stockl [29]	United States	Adult	Observational	Statin Prescribing, Statin Adherence	
2008	Hatfield [67]	United Kingdom	Adult	Observational	LDL-C, Statin Adherence	
2008	Coodley [88]	United States	Both	Observational	LDL-C	
2009	Stephenson [30]	United States	Adult and Child	Observational	LDL-C	
2009	Lima [40]	Brazil	Adult	Observational	LDL-C	
2009	Casebeer [59]	United States	Adult	Observational	Statin Adherence	
2010	Chen [75]	Taiwan	Adult	Observational	LDL-C	
2011	Gitt [49]	Germany	Adult	Observational	LDL-C	
2011	Chung [58]	Hong Kong	Adult	Observational	LDL-C	
2011	Schmittdiel [87]	United States	Adult	Observational	LDL-C	
2012	Aziz [68]	United States	Adult	Observational	LDL-C, Statin Prescribing	
2012	Farley [74]	United States	Adult	Observational	Statin Adherence	
2014	Clark [73]	United States	Adult and Child	Observational	Statin Adherence	
2014	Shoulders [84]	United States	Adult	Observational	LDL-C, Statin Prescribing	
2015	Vinker [26]	Israel	Adult	Observational	LDL-C, Statin Prescribing	
2016	Harrison [46]	United States	Adult	Observational	LDL-C, Statin Adherence	
2017	Andrews [64]	United States	Adult	Observational	Statin Adherence	
2018	Weng [22]	United Kingdom	Adult	Observational	LDL-C, Statin Prescribing	

### Implementation strategies

All implementation strategies except “provide interactive assistance” were used (Table 4). A total of 258 uses of strategies were identified across 86 studies. On average, each study utilized three strategies (SD 2.2, range 1–13). The most utilized strategies were “train and educate the stakeholders” (studies utilized strategies in this grouping 79 times), “support clinicians” (68), and “engage consumers” (47). The most utilized individual strategies were “intervene with patients and consumers to enhance uptake and adherence” (41), and “distribute educational materials” (41) (Additional file 1: Appendix 4). Implementation strategies often did not include key defining characteristics: temporality was reported 59% of the time, dose 52%, affected outcome 9%, and justification 6% (Table 2 provides a summary and Additional file 1: Appendix 5 provides a more detailed version).

**Table 4:** Summary of implementation strategies by strategy category

Strategy category	Strategies used per category	Total count within category	Meta-analysis total count within category
Use evaluative and iterative strategies	80% (8/10)	33	9
Support clinicians	80% (4/5)	68	20
Adapt and tailor to the context	75% (3/4)	4	2
Engage consumers	60% (3/5)	47	24
Train and educate the stakeholders	55% (6/11)	80	26
Change infrastructure	50% (4/8)	9	2
Develop stakeholder relationships	47% (8/17)	11	5
Utilize financial strategies	22% (2/9)	6	2
Provide interactive assistance	0% (0/4)	0	0

### Meta-analysis

**Fig. 2:**
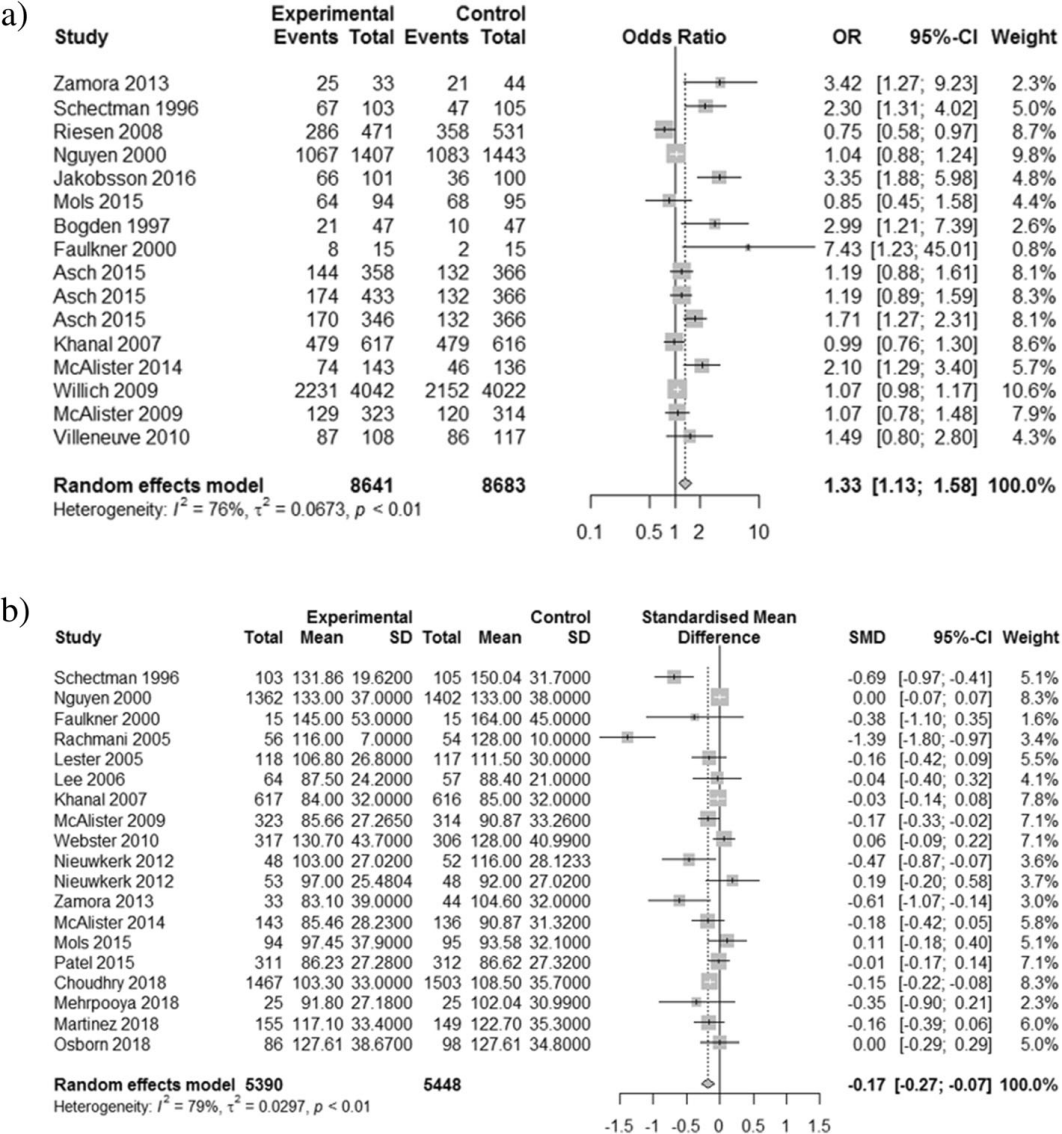
Forest plot of implementation strategies’ impact on LDL-C compared to control. **a** Achievement of target LDL-C. **b** Standardized mean difference in LDL-C

**Fig. 3:**
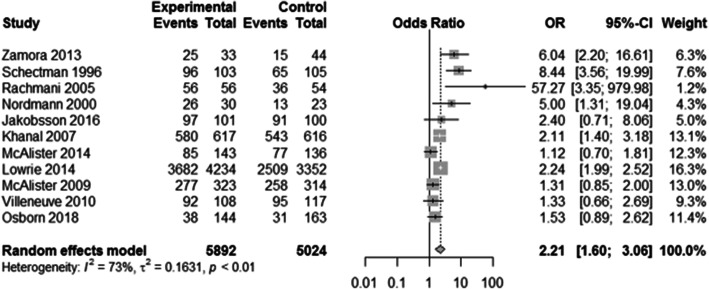
Forest plot of implementation strategies’ impact on statin prescribing compared to control

**Fig. 4:**
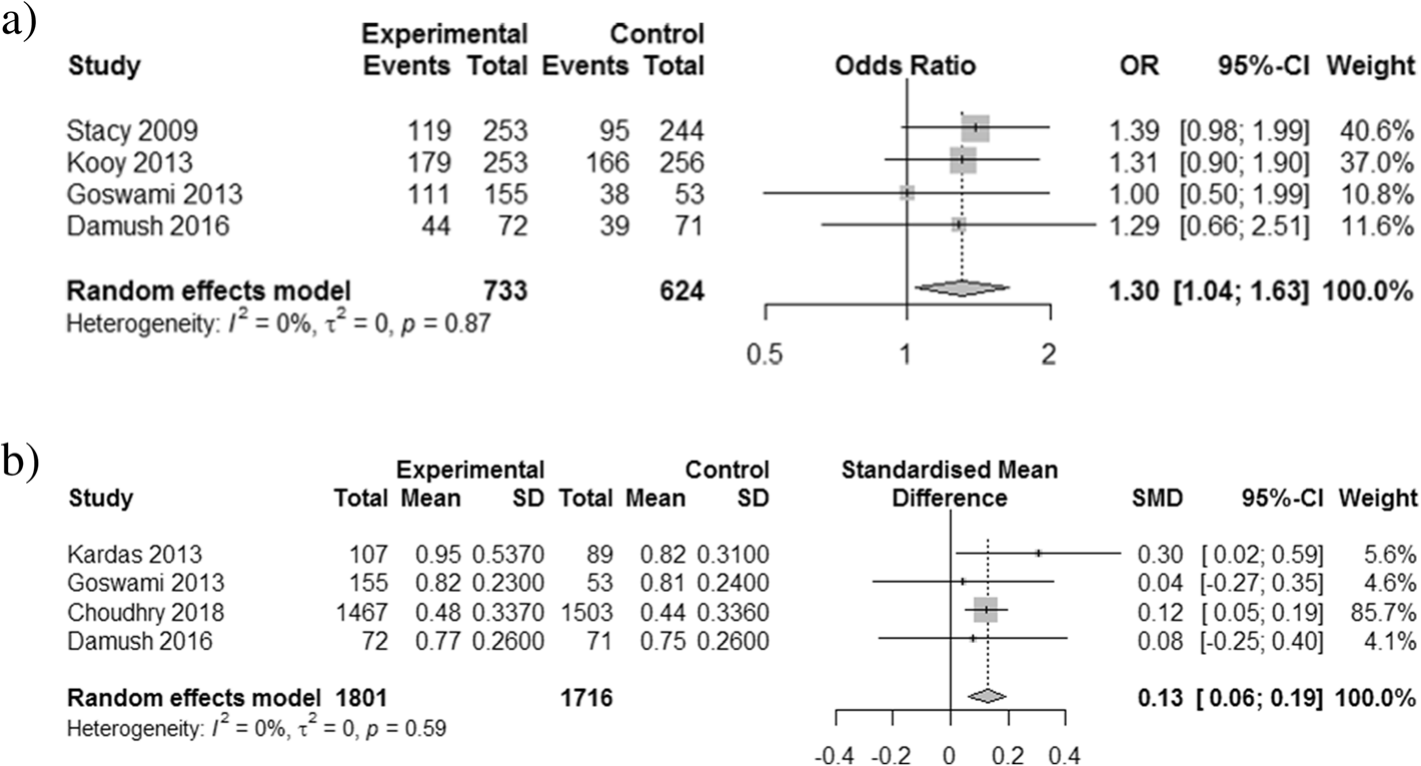
Forest plot of implementation strategies’ impact on statin adherence compared to control. **a** Medication possession ratio or portion of days covered > 80%. **b** Standardized mean difference in medication possession ratio or portion of days covered

Due to the large heterogeneity between studies, effectiveness outcomes (statin prescribing, statin adherence, and LDL-C) were only extracted from RCTs. Thirty-one trials reported at least one of the three outcomes of interest. The implementation strategies examined demonstrated: significantly reduced LDL-C (LDL-C reduction: SMD − 0.17, 95% CI − 0.27 to − 0.07, *p* = 0.0006; met LDL-C target: OR 1.33, 95% CI 1.13 to 1.58, *p* = 0.0008) (Fig. 2), increased rates of statin prescribing (OR 2.21, 95% CI 1.60 to 3.06, *p* < 0.0001) (Fig. 3), and improved statin adherence (PDC/MPR: SMD 0.13, 95% CI 0.06 to 0.19; *p* = 0.0002; ≥ 80% PDC/MPR: OR 1.30, 95% CI 1.04 to 1.63, *p* = 0.023) (Fig. 4). There was inconsistency across trials based on the outcome measured; statin prescribing (*I*^2^ = 73%), statin adherence (*I*^2^ = 0%), and LDL-C (*I*^2^ = 79% (LDL-C reduction) and 76% (met LDL-C targets)). Publication bias using the Egger’s test indicated no publication bias for statin prescribing (*p* = 0.63), statin adherence (*p* = 0.83 for SMD, *p* = 0.22 for OR), and potential publication bias for LDL-C (*p* = 0.08 for SMD, *p* = 0.01 for OR).

Although subgroup analyses were conducted for statin prescribing and LDL-C, there were not enough studies to conduct a subgroup analysis for statin adherence (Table 5). We identified a significant difference among studies published in 2013 or later for LDL-C measured as a binary outcome (OR 1.62, 95% CI1.19–2.19, *p* = 0.05). We also found a significant effect on LDL-C measured as a continuous variable when more than 2 implementation strategies were utilized (SMD − 0.38 95% CI − 0.67; − 0.09, *p* = 0.05). There was no significant effect in the between country analysis.

**Table 5:** Subgroup analyses

Study subgroup (number of studies)	Subgroup	Comparison group	*P* value for interaction
Odds ratio, (95% CI)			
*Statin prescribing* (11)			
More than 2 implementation strategies (6)	2.19 (1.32–3.63)	2.40 (1.43–4.06)	0.80
Study published in 2013 or later (5)	1.97 (1.29–3.01)	2.84 (1.41–5.74)	0.36
Conducted in the United States (2)	4.00 (1.03–15.50)	1.95 (1.33–2.84)	0.32
*LDL-C* (14)			
More than 2 implementation strategies (4)	1.53 (1.23–1.90)	1.20 (0.97–1.48)	0.12
Study published 2013 or later (5)	1.62 (1.19–2.19)	1.13 (0.95–1.35)	0.05
Conducted in the United States (5)	1.48 (1.12–1.95)	1.29 (1.03–1.61)	0.35
Standardized mean difference, (95% CI)			
*LDL-C* (17)			
More than 2 implementation strategies (6)	− 0.38 (− 0.67; − 0.09)	− 0.07 (− 0.15; − 0.01)	0.05
Study published in 2013 or later (8)	− 0.12 (− 0.21; v0.02)	− 0.23 (− 0.39; − 0.07)	0.24
Conducted in the United States (6)	− 0.20 (− 0.36; − 0.04)	− 0.17 (− 0.31; − 0.03)	0.79

Statin adherence was excluded because there were not enough studies to make a comparison

**Fig. 5:**
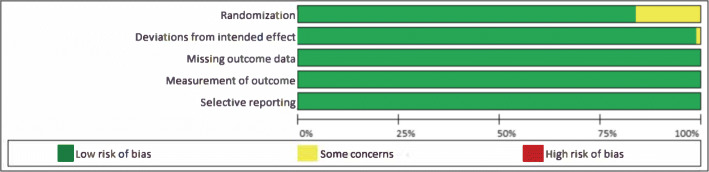
Risk of bias of RCTs included in the meta-analyses

Most studies were found to be at a low risk of bias (Fig. 5 and Additional file 1: Appendix 6); therefore, we did not conduct subgroup analyses based on the risk of bias.

## Discussion

### Our findings

In this review of implementation strategies regarding uptake of statins in hypercholesterolemia, we found that 38 different strategies were utilized to lower LDL-C, improve statin prescribing, and promote adherence. However, strategy components were not well defined and there was not a single strategy or group of strategies that demonstrated superior impact compared to others. Consistent with management of other diseases and conditions and literature from implementation science [106], we found evidence to support the use of multiple concurrent strategies; the use of three or more implementation strategies was associated with a greater reduction in LDL-C. We also found that studies published after 2012 had, on average, greater reductions in LDL-C through the use of the reported implementation strategies. While it cannot be definitely attributed, this could result from a better understanding of which strategies work best or could reflect a switch toward the utilization of high dose statin therapy. There was no difference in outcomes based on country where the study was conducted.

An important limitation of the many strategies described was incomplete definitions, limiting generalizability to other settings. Often, we were able to discern the actor, action, and action target but were unable to determine temporality, dose, implementation outcome affected, or justification. Without clear reporting of these factors, we are unable to interpret when these strategies should be used (temporality), how often (dosage), how the success of a specific strategy is measured (implementation outcomes affected), or when to justify the choice of a particular strategy (justification) to influence clinical practice. While the interventions appeared to be effective at increasing the utilization of statins and reducing LDL-C overall, the variable nature of the interventions studied and outcomes examined, the effectiveness of any specific strategy or set of strategies was unclear.

In addition, one category of strategies, “provide interactive assistance,” was not utilized in any of the studies included in the analysis. Among the strategies that were used, many were used in combination, but specific combinations were not used frequently enough to permit reliable subgroup analysis.

### Comparison with other studies

In the field of implementation science, there has recently been a desire to improve specification of implementation strategies utilized in practice and to develop standard language and definitions for reporting these implementation strategies [11, 14, 107]. This trend has led to the development of two implementation strategy taxonomies: the ERIC compilation [14], used in this study, and the Effective Practise and Organization of Care (EPOC) taxonomy [17]. Use of these taxonomies has allowed for consistent language in reporting implementation strategies and development of tailored compilations of strategies specific to certain disease states [108, 109]. Other systematic reviews of implementation strategies in other fields (i.e., intensive care setting and oral health) have found improved outcomes when multiple implementation strategies are used but have not been able to identify the groups of strategies most likely to produce the most favorable outcomes [110–112].

An investigation of enablers and barriers to treatment adherence in familial hypercholesterolemia found seven enablers for patients that could be used to develop new interventions and matched to implementation strategies we identified in our study [113]. These enablers were “other family members following treatment regime,” “commencement of treatment from a young age,” “parental responsibility to care for children,” “confidence in ability to successfully self-manage their condition,” “receiving formal diagnosis of familial hypercholesterolemia,” “practical resources and support for following lifestyle treatment,” and a “positive relationship with healthcare professionals” [113]. By linking the two most frequently used strategies identified in our systematic review “intervene with patients and consumers to enhance uptake and adherence” and “distribute educational materials,” with the enablers identified above, effective implementations strategies for statin utilization can be designed.

The sustainability of interventions to promote the uptake of guidelines when intervening at the clinician level has been limited in a variety of settings [114–116]. Specifically, in cardiovascular disease, a systematic review of interventions to improve uptake of heart failure medications saw an increase in guideline uptake but not improvement in clinical outcomes [117]. Similar findings have been found in hypertension [118]. However, the success of these interventions have been limited.

### Limitations and strengths

Our review is the first to comprehensively map the strategies used to increase utilization of statins among persons with hypercholesterolemia to the ERIC compilation. We chose to use ERIC due to a perceived better fit over alternatives (i.e. EPOC); however, we identified 7 studies (out of 208 identified) which could not be mapped to ERIC, exclusion of which could lead to missing important strategies. Other strengths include utilization of a medical librarian to conduct the search, searching of multiple databases which covered parts of the gray literature, and utilizing trained reviewers. Finally, we limited our search to studies in English with full-texts available. Thus, we may have missed studies not published in English or published in the gray literature (e.g., only conference abstract available in published literature) and be at risk for language bias [119] or publication bias [120]. While the Egger’s test suggested possible publication bias, we think that the risk of this is low due to our comprehensive search strategy. Further, while language bias is a possibility [119], few studies were excluded based on language so any potential impact is likely to be small.

### Suggestions for future research

Consistent strategies for reporting LDL-C would significantly improve the ability to assess efficacy of an intervention. Some studies used arbitrary cut-offs for LDL-C, some used absolute values, and others used thresholds published in cholesterol guidelines [121]. This led to difficulty in aggregating data across studies. Future studies should report absolute values of LDL-C to facilitate meta-analyses directed at change of LDL-C with intervention. Generating a core outcome set for trials in hypercholesterolemia would facilitate meta-analyses and ensure all relevant outcomes are consistently measured [122]. Ideally, these studies should be registered and included in a meta-analysis in a prospective manner [123].

Clarity in the terminology, definition, and description of implementation strategies by researchers would help translation and replication of efforts. Completely reporting implementation strategies facilitates interpretation of results as well as facilitating reproducibility and scalability [11]. The field of implementation science offers guidance on how to name and report these strategies [11]. Even though this study was unable to identify a single or gold standard approach to improving statin therapy for hypercholesterolemia disorders, it provides examples of many different approaches that have some impact on outcomes relevant to care. In this way, this study provides a roadmap for future implementation to better define implementation strategies and to rigorously define and test the outcomes associated with those strategies. More guidance will be needed on the impact of strategies in different healthcare settings, because different strategies may work better in different healthcare settings so these idiosyncrasies need to be understood.

## Conclusion

Implementation strategies to improve the uptake of statins among patients with hypercholesterolemia exist but they are poorly reported and generalizability is limited. While these strategies lowered LDL-C and improved adherence, significant heterogeneity made assessment of the comparative effectiveness of strategies difficult. Future studies for increasing the utilization of statins among patients with hypercholesterolemia should more clearly define strategies used, prospectively test comparative effectiveness of different strategies, and use standardized efficacy endpoints.

## Supplementary Information

**Additional file 1: Appendix 1**. Statin uptake search strategy. **Appendix 2**. Excluded full text articles and rationale. **Appendix 3**. Detailed study demographics. **Appendix 4**. Count of implementation strategy organized by category and strategy. **Appendix 5**. Detailed Proctor’s framework description of each strategy. **Appendix 6**. Risk of bias

## Data Availability

All data generated or analyzed during this study are included in this published article and its supplementary information files.

## Abbreviations

CIs: Confidence intervals
ERIC: Expert Recommendations for Implementing Change
LDL-C: Low density lipoprotein cholesterol
ORs: Odds ratios
PRISMA: Preferred Reporting Items for Systematic Reviews and Meta-analyses
SMDs: Standardized mean differences
RCTs: Randomized clinical trails
