# Supplementary material for: Implementation strategies to improve statin utilization in individuals with hypercholesterolemia: a systematic review and meta-analysis
Source: Implement Sci. 2021 Apr 13;16:40. doi: 10.1186/s13012-021-01108-0 (PMC8045284; doi:10.1186/s13012-021-01108-0)
Supplement: Supplementary file 1 — Additional file 1: Appendix 1. Statin uptake search strategy. Appendix 2. Excluded full text articles and rationale. Appendix 3. Detailed study demographics. Appendix 4. Count of implementation strategy organized by category and strategy. Appendix 5. Detailed Proctor’s framework description of each strategy. Appendix 6. Risk of bias [file 13012_2021_1108_MOESM1_ESM.docx]

Additional file 1:

Appendix 1. Statin uptake search strategy

Appendix 2. Excluded full text articles and rationale

Appendix 3. Detailed study demographics

Appendix 4. Count of implementation strategy organized by category and strategy

Appendix 5. Detailed Proctor’s framework description of each strategy

Appendix 6. Risk of bias

Appendix 1. Statin uptake search strategy

**Fully Reproducible Copy and Paste searches:**

Embase

Date Searched: 10/18/2018

Applied Database Supplied Limits: n/a

Number of Results: 31,632

Full Search Strategy:

('hypercholesterolemia'/exp OR 'familial hypercholesterolemia'/exp OR hypercholesterolemia:ti,ab,kw OR cholesteremia:ti,ab,kw OR cholesterinemia:ti,ab,kw OR cholesterolemia:ti,ab,kw OR hypercholesteremia:ti,ab,kw OR hypercholesterinaemia:ti,ab,kw OR hypercholesterinemia:ti,ab,kw OR hypercholesterolaemia:ti,ab,kw OR (('high cholesterol' NEAR/1 level*):ti,ab,kw) OR ((elevated NEAR/1 cholesterol*):ti,ab,kw) OR 'hyperlipidemia'/exp OR 'familial hyperlipemia'/exp OR hyperlipemia*:ti,ab,kw OR hyperlipaemia:ti,ab,kw OR hyperlipemia:ti,ab,kw OR hyperlipidaemia:ti,ab,kw OR hyperlipidaemias:ti,ab,kw OR hyperlipidemias:ti,ab,kw OR hyperlipidemic:ti,ab,kw OR lipaemia:ti,ab,kw OR lipemia:ti,ab,kw OR lipidaemia:ti,ab,kw OR lipidemia:ti,ab,kw) AND ('hydroxymethylglutaryl coenzyme a reductase inhibitor'/exp OR 'hydroxymethylglutaryl coenzyme a reductase inhibitor':ti,ab,kw OR 'hydroxymethylglutaryl-coa inhibitors':ti,ab,kw OR 'hydroxymethylglutaryl-coenzyme a inhibitors':ti,ab,kw OR 'hmg coa reductase inhibitor':ti,ab,kw OR 'hmg coenzyme a reductase inhibitor':ti,ab,kw OR 'hmg coa reductase inhibitors':ti,ab,kw OR 'hydroxymethylglutaryl coa reductase inhibitors':ti,ab,kw OR 'hydroxymethylglutaryl-coa reductase inhibitors':ti,ab,kw OR statin:ti,ab,kw OR statins:ti,ab,kw OR vastatin:ti,ab,kw)

Ovid Medline

Date Searched: 10/22/2018
Applied Database Supplied Limits: n/a
Number of Results: 11,819

Full Search Strategy:

(Exp "Hypercholesterolemia"/ OR (hypercholesterolemia OR cholesteremia OR cholesterinemia OR cholesterolemia OR hypercholesteremia OR hypercholesterinaemia OR hypercholesterinemia OR hypercholesterolaemia).mp. OR (High Cholesterol adj1 Level*).mp. OR (Elevated adj1 Cholesterol*).mp. OR exp "Hyperlipidemias"/ OR exp "Hyperlipidemia, Familial Combined"/ OR (hyperlipemia* OR hyperlipaemia OR hyperlipemia OR hyperlipidaemia OR hyperlipidaemias OR hyperlipidemias OR hyperlipidemic OR lipaemia OR lipemia OR lipidaemia OR lipidemia).mp.) AND (exp Hydroxymethylglutaryl-CoA Reductase Inhibitors/ OR (hydroxymethylglutaryl coenzyme a reductase inhibitor OR Hydroxymethylglutaryl-CoA Inhibitors OR Hydroxymethylglutaryl-Coenzyme A Inhibitors OR hmg coa reductase inhibitor OR hmg coa reductase inhibitors OR hmg coenzyme a reductase inhibitor OR hmg coa reductase inhibitors OR hydroxymethylglutaryl coa reductase inhibitors OR hydroxymethylglutaryl-coa reductase inhibitors OR statin OR statins OR vastatin).mp.)

Scopus

Date Searched: 10/22/2018
Applied Database Supplied Limits: n/a
Number of Results: 17,238

Full Search Strategy:

((TITLE-ABS-KEY (hypercholesterolemia OR cholesteremia OR cholesterinemia OR cholesterolemia OR hypercholesteremia OR hypercholesterinaemia OR hypercholesterinemia OR hypercholesterolaemia OR “High Cholesterol Level*” OR “Elevated Cholesterol*”)) OR (TITLE-ABS-KEY(hyperlipemia* OR hyperlipaemia OR hyperlipemia OR hyperlipidaemia OR hyperlipidaemias OR hyperlipidemias OR hyperlipidemic OR lipaemia OR lipemia OR lipidaemia OR lipidemia))) AND ((TITLE-ABS-KEY(“hydroxymethylglutaryl coenzyme a reductase inhibitor” OR “Hydroxymethylglutaryl-CoA Inhibitors” OR “Hydroxymethylglutaryl-Coenzyme A Inhibitors” OR “hmg coa reductase inhibitor” OR “hmg coa reductase inhibitors” OR “hmg coenzyme a reductase inhibitor” OR “hmg coa reductase inhibitors” OR “hydroxymethylglutaryl coa reductase inhibitors” OR “hydroxymethylglutaryl-coa reductase inhibitors” OR statin OR statins OR vastatin)))

Cochrane Library

Date Searched: 10/22/2018
Applied Database Supplied Limits: n/a
Number of Results:

CDSR: 18

CENTRAL: 2671

Full Search Strategy:

([mh "Hypercholesterolemia"] OR (hypercholesterolemia OR cholesteremia OR cholesterinemia OR cholesterolemia OR hypercholesteremia OR hypercholesterinaemia OR hypercholesterinemia OR hypercholesterolaemia):ti,ab,kw OR (High Cholesterol NEAR/1 Level*):ti,ab,kw OR (Elevated NEAR/1 Cholesterol*):ti,ab,kw OR [mh "Hyperlipidemias"] OR [mh "Hyperlipidemia, Familial Combined"] OR (hyperlipemia* OR hyperlipaemia OR hyperlipemia OR hyperlipidaemia OR hyperlipidaemias OR hyperlipidemias OR hyperlipidemic OR lipaemia OR lipemia OR lipidaemia OR lipidemia):ti,ab,kw) AND ([mh "Hydroxymethylglutaryl-CoA Reductase Inhibitors"] OR (“hydroxymethylglutaryl coenzyme a reductase inhibitor” OR “Hydroxymethylglutaryl-CoA Inhibitors” OR “Hydroxymethylglutaryl-Coenzyme A Inhibitors” OR “hmg coa reductase inhibitor” OR “hmg coa reductase inhibitors” OR “hmg coenzyme a reductase inhibitor” OR “hmg coa reductase inhibitors” OR “hydroxymethylglutaryl coa reductase inhibitors” OR “hydroxymethylglutaryl-coa reductase inhibitors” OR statin OR statins OR vastatin):ti,ab,kw)

ClinicalTrials.gov

Date Searched: 10/22/2018

Number of Results: 750

(hypercholesterolemia OR hyperlipemia) AND (“hydroxymethylglutaryl coenzyme a reductase inhibitor” OR statins)

Appendix 2. Excluded full text articles and rationale

Articles could have had multiple reasons for exclusion but only one was arbitrarily chosen for this report.

| **Reason for exclusion:**  **Study design** | |
| --- | --- |
| Ref ID: 1283 | Virani SS, Woodard LD, Chitwood SS, Landrum CR, Urech TH, Wang D, Murawsky J, Ballantyne CM, Petersen LA. Frequency and correlates of treatment intensification for elevated cholesterol levels in patients with cardiovascular disease. *American Heart Journal*. 2011; 162:725-732.e1 |
| Ref ID: 1508 | Van Wyk JT, Picelli G, Dieleman JP, Mozaffari E, Kramarz P, Van Wijk MAM, Van Der Lei J, Sturkenboom MCJM. Management of hypertension and hypercholesterolaemia in primary care in the Netherlands. *Current Medical Research and Opinion*. 2005; 21:839-848 |
| Ref ID: 1598 | Van Delden XM, Huijgen R, Wolmarans KH, Brice BC, Barron JK, Blom DJ, Marais AD. LDL-cholesterol target achievement in patients with heterozygous familial hypercholesterolemia at Groote Schuur Hospital: Minority at target despite large reductions in LDL-C. *Atherosclerosis*. 2018; 277:327-333 |
| Ref ID: 1932 | Travis Gossey J, Whitney SN, Crouch MA, Jibaja-Weiss ML, Zhang H, Volk RJ. Promoting knowledge of statins in patients with low health literacy using an audio booklet. *Patient Preference and Adherence*. 2011; 5:397-403 |
| Ref ID: 1942 | Tran JN, Caglar T, Stockl KM, Lew HC, Solow BK, Chan PS. Impact of the new ACC/AHA guidelines on the treatment of high blood cholesterol in a managed care setting. *American Health and Drug Benefits*. 2014; 7:430-443 |
| Ref ID: 2587 | Tait AR, Voepel-Lewis T, Brennan-Martinez C, McGonegal M, Levine R. Using animated computer-generated text and graphics to depict the risks and benefits of medical treatment. *American Journal of Medicine*. 2012; 125:1103-1110 |
| Ref ID: 2654 | Sy FZ, Choe HM, Kennedy DM, Standiford CJ, Parsons DM, Bruhnsen KD, Stevenson JG, Bernstein SJ. Moving from A to Z: Successful implementation of a statin switch program by a large physician group. *American Journal of Managed Care*. 2009; 15:233-240 |
| Ref ID: 3543 | Simons LA, Ortiz M, Calcino G. Persistence with a single pill versus two pills of amlodipine and atorvastatin: The Australian experience, 2006-2010. *Medical Journal of Australia*. 2011; 195:134-137 |
| Ref ID: 6493 | Oster G, Borok GM, Menzin J, Heyse JF, Epstein RS, Quinn V, Benson V, James Dudl R, Epstein A. A randomized trial to assess effectiveness and cost in clinical practice: Rationale and design of the cholesterol reduction intervention study (CRIS). *Controlled Clinical Trials*. 1995; 16:3-16 |
| Ref ID: 7013 | Ng C, Chung P, Toderika Y, Cheng-Lai A. valuation of Adherence to current guidelines for treatment of hyperlipidemia in adults in an outpatient setting. American Journal of Health-System Pharmacy. 2016. 73:S133-S140 |
| Ref ID: 8443 | Martin SC, Viljoen A. The value of a specialist lipid clinic. *International Journal of Clinical Practice*. 2008; 62:961-966 |
| Ref ID: 8499 | Mark L, Paragh G, Karadi I, Reiber I, Pados G, Kiss Z. How can we further improve the LDL-cholesterol target level achievement rate based on the Hungarian MULTI GAP 2011 study results and considering the new European dyslipidemia guidelines? *Archives of Medical Science*. 2012; 8:608-613 |
| Ref ID: 8812 | MacInnes JA, Salkovskis PM, Wroe A, Hope T. Helping patients to reach decisions regarding their treatment: Do 'non-directive' approaches cause systematic bias? *British journal of health psychology*. 2015; 20:877-888 |
| Ref ID: 8815 | Maciejewski ML, Bryson CL, Perkins M, Blough DK, Cunningham FE, Fortney JC, Krein SL, Stroupe KT, Sharp ND, Liu CF. ncreasing copayments and adherence to diabetes, hypertension, and hyperlipidemic medications. *The American journal of managed care*. 2010; 16:e20-34 |
| Ref ID: 8816 | Maciejewski M, Wansink D, Farley J. Turning the tide? Medication adherence after copayment reductions. *Journal of General Internal Medicine*. 2010; 25:S418-S419 |
| Ref ID: 8996 | Lopez-Carmona D, Bernal-Lopez MR, Mancera-Romero J, Jansen-Chaparro S, Portales-Fernandez I, Baca-Osorio AJ, Perez-Gonzalez R, Tinahones FJ, Gomez-Huelgas R. Compliance with cardiovascular drug prevention measures in a general population: The Multidisciplinary Intervention in Primary Care (IMAP) study. *European Journal of Preventive Cardiology*. 2012; 19:1074-1081 |
| Ref ID: 10211 | Koren MJ, Hunninghake DB. Clinical outcomes in managed-care patients with coronary heart disease treated aggressively in lipid-lowering disease management clinics: The alliance study. *Journal of the American College of Cardiology*. 2004; 44:1772-1779 |
| Ref ID: 10835 | Kaya H, Beton O, Yilmaz MB. How to increase level of patients' awareness regarding the importance of statins despite the influence of the media? *International Journal of Cardiology*. 2016; 207:164 |
| Ref ID: 11151 | Kahoul R, Gueyffier F, Amsallem E, Haugh M, Marchant I, Boissel FH, Boissel JP. Comparison of an effect-model-law-based method versus traditional clinical practice guidelines for optimal treatment decision making: Application to statin treatment in the French population. *Journal of the Royal Society Interface*. 2014; 11(100):20140867. |
| Ref ID: 12542 | Harrison TN, Scott RD, Cheetham TC, Chang SC, Hsu JWY, Wei R, Ling Grant DS, Boklage SH. Romo-LeTourneau V, Reynolds K. Trends in Statin Use 2009–2015 in a Large Integrated Health System: Pre- and Post-2013 ACC/AHA Guideline on Treatment of Blood Cholesterol. *Cardiovascular Drugs and Therapy*. 2018; 32:397-404 |
| Ref ID: 12569 | Harmsen CG, Jarbøl DE, Nexøe J, Støvring H, Gyrd-Hansen D, Nielsen JB, Edwards A, Kristiansen IS. Impact of effectiveness information format on patient choice of therapy and satisfaction with decisions about chronic disease medication: the "Influence of intervention Methodologies on Patient Choice of Therapy (IMPACT)" cluster-randomised trial in general practice. *BMC health services research*. 2013; 13:76 |
| Ref ID: 12882 | Gulayin P, Irazola V, Lozada A, Chaparro M, Santero M, Gutierrez L, Poggio R, Beratarrechea A, Rubinstein A. Educational intervention to improve effectiveness in treatment and control of patients with high cardiovascular risk in low-resource settings in Argentina: Study protocol of a cluster randomised controlled trial. *BMJ Open*. 2017;7(1):e014420. |
| Ref ID: 13070 | Grace KA, Swiecki J, Hyatt R, Gibbs H, Jones DL, Sheikh M, Spain J, Maneval KW, Viola R, Taylor AJ. Implementation of a therapeutic-interchange clinic for HMG-CoA reductase inhibitors. *American Journal of Health-System Pharmacy*. 2002; 59:1077-1082 |
| Ref ID: 13447 | Gibson TB, Mark TL, Axelsen K., Baser O, Rublee DA, McGuigan KA. Impact of statin copayments on adherence and medical care utilization and expenditures. *American Journal of Managed Care*. 2006; 12:SP11-SP19 |
| Ref ID: 13448 | Gibson TB, Mark TL, McGuigan KA, Axelsen K, Wang S. The effects of prescription drug copayments on statin adherence. *American Journal of Managed Care*. 2006; 12:509-517 |
| Ref ID: 14290 | Farnier M, Civeira F, Descamps O, Kawashiri M, Grigore L, Vohnout B. How to implement clinical guidelines to optimise familial hypercholesterolaemia diagnosis and treatment. *Atherosclerosis Supplements*. 2017; 26:25-35 |
| Ref ID: 14393 | Fàbregas M, Berges I, Fina F, Hermosilla E, Coma E, Méndez L, Medina M, Calero S, Serrano E, Morros R, Monteagudo M, Bolíbar B. Effectiveness of an intervention designed to optimize statins use: a primary prevention randomized clinical trial. *BMC family practice*. 2014; 15:135 |
| Ref ID: 15497 | David Bradford W, Kleit AN, Nietert PJ, Ornstein S. Effects of direct-to-consumer advertising of hydroxymethylglutaryl coenzyme a reductase inhibitors on attainment of LDL-C goals. *Clinical Therapeutics*. 2006; 28:2105-2118 |
| Ref ID: 16215 | Cheng CWR, Woo KS, Chan JCN, Tomlinson B, You JHS. Assessing adherence to statin therapy using patient report, pill count, and an electronic monitoring device. *American Journal of Health-System Pharmacy*. 2005; 62:411-415 |
| Ref ID: 16368 | Chang HY, Murimi I, Daubresse M, Qato DM, Emery SL, Alexander GC. Effect of Direct-to-Consumer Advertising on Statin Use in the United States. *Medical Care*. 2017; 55:759-764 |
| Ref ID: 18743 | Amariles P, Sabater-Hernández D, García-Jiménez E, Rodríguez-Chamorro MÁ, Prats-Más R, Marín-Magán F, Galán-Ceballos JA, Jiménez-Martín J, José Faus M. Effectiveness of dader method for pharmaceutical care on control of blood pressure and total cholesterol in outpatients with cardiovascular disease or cardiovascular risk: EMDADER-CV randomized controlled trial. *Journal of Managed Care Pharmacy*. 2012; 18:311-323 |
| Ref ID: 20195 | Duncan MC, Castle SS, Streetman DS. Effect of tablet splitting on serum cholesterol concentrations. *Annals of Pharmacotherapy*. 2002; 36:205-209 |
| Ref ID: 21028 | Taylor AM, Bingham J, Schussel K, Axon DR, Dickman DJ, Boesen K, Martin R, Warholak TL. Integrating innovative telehealth solutions into an interprofessional team-delivered chronic care management pilot program. *Journal of Managed Care and Specialty Pharmacy*. 2018; 24:813-818 |
| Ref ID: 21334 | Atlas SJ, Jernigan M, Ashburner JM, Chang J, Borowsky LH, Chang Y, Grant RW. The medication metronome: A health it system to improve medication management and laboratory monitoring for chronic diseases. *Journal of General Internal Medicine*. 2014; 29:S230 |
| Ref ID: 21687 | Lederle FA, Rogers EM. Lowering the cost of lowering the cholesterol: A formulary policy for lovastatin. *Journal of General Internal Medicine*. 1990; 5:459-463 |
| Ref ID: 23616 | Schoen MD, Didomenico RJ, Connor SE, Dischler JE, Bauman JL. Impact of the cost of prescription drugs on clinical outcomes in indigent patients with heart disease. *Pharmacotherapy*. 2001; 21:1455-1463 |
| Ref ID: 24941 | Sandhoff BG, Nies LK, Olson KL, Nash JD, Rasmussen JR, Merenich JA. Clinical pharmacy cardiac risk service for managing patients with coronary artery disease in a health maintenance organization. *American Journal of Health-System Pharmacy*. 2007; 64:77-84 |
| Ref ID: 30018 | Mehler PS, Krantz MJ, Lundgren RA, Estacio RO, MacKenzie TD, Petralia L, Hiatt WR. Bridging the quality gap in diabetic hyperlipidemia: A practice-based intervention. *American Journal of Medicine*. 2005; 118:1414.e13-1414.e19 |
| Ref ID: 30986 | Israel EN, Farley TM, Farris KB, Carter BL. Underutilization of cardiovascular medications: Effect of a continuity-of-care program. *American Journal of Health-System Pharmacy*. 2013; 70:1592-1600 |
| Ref ID: 31815 | Pokharel Y, Steinberg L, Chan W, Akeroyd JM, Jones PH, Nambi V, Nasir K, Petersen L, Ballantyne CM, Virani SS. Case-based educational intervention to assess change in providers' knowledge and attitudes towards the 2013 American College of Cardiology/American Heart Association Cholesterol Management Guideline. *Atherosclerosis*. 2016; 246:115-120 |
| Ref ID: 32600 | Mainous Iii AG, Baker R, Everett CJ, King DE. Impact of a policy allowing for over-the-counter statins. *Quality in Primary Care*. 2010; 18:301-306 |
| Ref ID: 32601 | Lowrie R, Morrison J, McConnachie A. A cluster randomised controlled trial of pharmacist led Statin Outreach Support (SOS) in primary care: Design and baseline characteristics. *Contemporary Clinical Trials*. 2010; 31:303-311 |
| Ref ID: 32633 | Weinstock RS, Izquierdo R, Goland R, Palmas W, Teresi JA, Eimicke JP, Shea S. Lipid treatment in ethnically diverse underserved older adults with diabetes mellitus: Statin use, goal attainment, and health disparities in the informatics for diabetes education and telemedicine project*. Journal of the American Geriatrics Society*. 2010; 58:401-402 |
| Ref ID: 32678 | Dalmau R, Boira M, Aguilar C, López C, Rodríguez D, Gentille D, Bofill D, Diogene E, Pepió JM. Lipid-lowering drugs in ischaemic heart disease: A quasi-experimental uncontrolled before-and-after study of the effectiveness of clinical practice guidelines. *BMC Cardiovascular Disorders*. 2011; 11:47. |
| Ref ID: 32795 | Colvine K, Kerr AJ, McLachlan A, Gow P, Kumar S, Ly J, Wiltshire C, Robinson E, Dalbeth N. Cardiovascular disease risk factor assessment and management in gout: An analysis using guideline-based electronic clinical decision support. *New Zealand Medical Journal.* 2008; 121(1285):73-81. |
| Ref ID: 32822 | Olson KL, Delate T, Rasmussen J, Humphries TL, Merenich JA. Outcomes of patients discharged from pharmacy-managed cardiovascular disease management. *American Journal of Managed Care*. 2009; 15:497-503 |
| Ref ID: 32834 | Brass EP, Vassil T, Replogle A, Hwang P, Rusche S, Shiffman S, Levine JG. Can Consumers Self-Select for Appropriate Use of an Over-the-Counter Statin? The Self Evaluation of Lovastatin to Enhance Cholesterol Treatment Study. *American Journal of Cardiology*. 2008; 101:1448-1455 |
| Ref ID: 33226 | Thiebaud P, Patel BV, Nichol MB, Berenbeim DM. The effect of switching on compliance and persistence: The case of statin treatment. *American Journal of Managed Care*. 2005; 11:670-674 |
| Ref ID: 33591 | Williams ML, Morris Ii MT, Ahmad U, Yousseff M, Li W, Ertel N. Racial differences in compliance with NCEP-II recommendations for secondary prevention at a veterans affairs medical center. *Ethnicity and Disease*. 2002; 12:S1-58-S1-62 |
| Ref ID: 34419 | Egan BM, Sutherland SE, Childers WF, Dahlheimer RM, Helmrich GA, Lapeyrolerie DA, Markle N, Murphy DW, Simmons L, Davis RA, Tilkemeier P, Sinopoli A. Comparative impact of implementing the 2013 or 2014 cholesterol guideline on vascular events in a quality improvement network. *Therapeutic Advances in Cardiovascular Disease*. 2016; 10:56-66 |
| Ref ID: 34998 | Martikainen JA, Soini E, Paulsson T. Cost-effectiveness of single agent, uptitration and switching statin treatment strategies for lipid lowering in Sweden. *Current Medical Research & Opinion*. 2010; 26:389-96 |
| Ref ID: 36296 | L'Italien G, Ford I, Norrie J, LaPuerta P, Ehreth J, Jackson J, Shepherd, J. The cardiovascular event reduction tool (CERT)--a simplified cardiac risk prediction model developed from the West of Scotland Coronary Prevention Study (WOSCOPS). *American Journal of Cardiology*. 2000; 85:720-4 |
| **Reason for exclusion:**  **No strategy category** | |
| Ref ID: 425 | Wu NQ, Guo YL, Ye P, Chen H, Li YF, Hua Q, Zhu CG, Gao Y, Qing P, Li XL, Wang Y, Liu G, Dong Q, Li JJ. Statins usage and target achievement of LDL-C level in Chinese patients with coronary artery disease impacted by 2013 ACC/AHA cholesterol guideline. *IJC Metabolic and Endocrine*. 2017; 14:33-37 |
| Ref ID: 10080 | Kozela M, Szafraniec K, Broda G, Cichocka I, Drygas W, Ga̧sior Z, Grodzicki T, Janion M, Szpak A, Wizner B, Wolfshaut-Wolak R, Zdrojewski T, Paja̧k A. Detection and treatment of hypercholesterolemia in primary health care: Results of the POLKARD program of the Ministry of Health of the Republic of Poland. *Polskie Archiwum Medycyny Wewnetrznej*. 2012; 122:154-161 |
| Ref ID: 16364 | Chang KCM, Lee JT, Vamos EP, Soljak M, Johnston D, Khunti K, Majeed A, Millett C. Impact of the National Health Service Health Check on cardiovascular disease risk: A difference-in-differences matching analysis. *CMAJ*. 2016; 188:E228-E238 |
| Ref ID: 20658 | Forster AS, Burgess C, Dodhia H, Fuller F, Miller J, McDermott L, Gulliford MC. Do health checks improve risk factor detection in primary care? Matched cohort study using electronic health records. *Journal of public health (Oxford, England).* 2016; 38:552-559 |
| Ref ID: 33014 | Wei L, MacDonald TM, Watson AD, Murphy MJ. Effectiveness of two statin prescribing strategies with respect to adherence and cardiovascular outcomes: Observational study. *Pharmacoepidemiology and Drug Safety*. 2007; 16:385-392 |
| Ref ID: 34970 | Hussein MA, Chapman RH, Benner JS, Tang SS, Solomon HA, Joyce A, Foody JM. Does a single-pill antihypertensive/lipid-lowering regimen improve adherence in US managed care enrolees? A non-randomized, observational, retrospective study. *American Journal of Cardiovascular Drugs*. 2010; 10:193-202 |
| Ref ID: 35500 | McKenney JM, Davidson MH, Saponaro J, Thompson PD, Bays HE. Use of a treatment algorithm to achieve NCEP ATP III goals with atorvastatin*. Journal of Cardiovascular Pharmacology*. 2005; 46:594-9 |
| **Reason for exclusion:**  **Non-statin** | |
| Ref ID: 8801 | Madejski RM, Madejski TJ. Cholesterol screening in a community pharmacy. *Journal of the American Pharmaceutical Association*. 1996; 36:243-248 |
| Ref ID: 25758 | Chiu CC, Wu SS, Lee PY, Huang YC, Tan TY, Chang KC. Control of modifiable risk factors in ischemic stroke outpatients by pharmacist intervention: An equal allocation stratified randomized study*. Journal of Clinical Pharmacy and Therapeutics*. 2008; 33:529-535 |
| **Reason for exclusion:**  **No full text** | |
| Ref ID: 546 | Wing L, Bielinska A, Knight K. Improving rates of statin prescription in vascular surgery patients. *QJM*. 2011; 104:362-363 |
| Ref ID: 827 | Weinstein E, Nelson I. Using poopulation management strategies to improve lipid management in high risk patients. *Journal of General Internal Medicine*. 2016; 31:S920 |
| Ref ID: 3136 | Spyropoulos J, Chatterjee P, LaCouture M. Lipid management in clinical practice: Optimizing outcomes through dual physician-patient education. *Journal of Clinical Lipidology*. 2016; 10:684-685 |
| Ref ID: 3138 | Wing L, Bielinska A, Knight K. Improving rates of statin prescription in vascular surgery patients. *QJM*. 2011; 104:362-363 |
| Ref ID: 4173 | Schlyter M, Ogmundsottir Michelsen H, Sjolin I, Hag E, Hagstrom E, Nilsson L, Kiessling A, Held C, Schiopu A, Zaman MJ, Leosdottir M. Myocardial infarction patients more often reach treatment goals for low-density lipoprotein at centres where cardiac rehabilitation nurses adjust statins-the Perfect-CR study. *European Heart Journal.* 2017; 38:519 |
| Ref ID: 4867 | Rosenblatt H, Higginbotham S, Skomo M, Stewart A. Pharmacist interventions using the health belief model: Impact on statin adherence. *Journal of the American Pharmacists Association*. 2012; 52:205 |
| Ref ID: 7393 | Müller-Nordhorn Ja, Englert H, Wegscheider K, Völler H, Sonntag F, Meyer-Sabellek W, Windler E, Katus HA, Willich SN. Effect of an adherence programme on cardiovascular events in high-risk patients with hypercholesterolemia. *Atherosclerosis Supplements*. 2011; 12:117 |
| Ref ID: 7753 | Mitchell M, Dunn J. Evaluation of an interactive voice response (IVR) statin adherence program. *Journal of Managed Care Pharmacy*. 2011; 17:558-559 |
| Ref ID: 8336 | Masnaghetti S, Proserpio C, Bestetti L, Bertipaglia D, Maslowsky F, Guzzetti D, Braga SS, Pedretti R. Efficacy of cardiovascular primary prevention: 6 Months follow-up results. *High Blood Pressure and Cardiovascular Prevention*. 2013; 20:100 |
| Ref ID: 8485 | Markovitz AA, Holleman R, Klamerus ML, Hofer T, Kerr EA, Sussman J. The impact of the 2013 national cholesterol treatment guideline in the VA: An interrupted time series analysis. *Journal of General Internal Medicine*. 2017; 32:S345-S346 |
| Ref ID: 8517 | Maria Usman Khan MU, Khan UA, Adeeb F, Maher E, Devlin J, Fraser A. Indications for lowering LDL Cholesterol in Rheumatoid Arthritis: An unrecognized problem. European Journal of Preventive Cardiology. 2018. 25:S58 |
| Ref ID: 9511 | Lee JH, Longmore R, Markiewicz R, McGhie AI, O'Keefe JH, Hsu BL, Kennedy K, Thompson RC, Bateman TM, Bybee KA. Evidence of coronary calcium in patients with normal positron emission tomography myocardial perfusion imaging scans likely to influence physicians to initiate statins or recommend optimal medical therapy. *Journal of the American College of Cardiology*. 2009; 53:A296 |
| Ref ID: 10089 | Kouz S, Constance C, Rampakakis E, Psaradellis E, Sampalis J, De Carolis E. Real-life implementation of a LDL-C treat-to-target strategy in the management of hyperlipidemia in high CVD risk patients in Québec. *Canadian Journal of Cardiology*. 2012; 28:S244-S245 |
| Ref ID: 10915 | Katada J, Hirai M, Takahashi S. A single-pill antihypertensive/lipid-lowering regimen improves adherence to treatment in general Japanese patients [abstract]. *Circulation*. 2012; 126(21):A11742. https://www.ahajournals.org/doi/abs/10.1161/circ.126.suppl_21.a11742. Accessed November 12, 2019. |
| Ref ID: 12242 | Ho KT, Hameed RS, Molina JD, Razakjr A, Ong HY, Heng BH. Good cholesterol control in a large disease management initiative: Lessons from the LIVE program. *European Heart Journal*. 2009; 30:440 |
| Ref ID: 14329 | Farid W, Crispin G, Sengotta K, Southwell L, Watts G. A family support group for Familial Hypercholesterolaemia: Experiences in Western Australia. *Heart Lung and Circulation*. 2011; 20:S241 |
| Ref ID: 14846 | Dresser GK, Nelson SAE, Mahon JL, Zou GY, Vandervoort MK, Wong CJ, Feagan BG, Feldman RD. Evaluation of a treatment algorithm using combination therapy for the management of patients with hypertension and hypercholesterolemia (STITCH2). *Journal of Clinical Hypertension*. 2011; 13:A22-A23 |
| Ref ID: 14876 | Dougherty T. Effect of motivational interviewing on medication adherence in a community pharmacy: Potential implications for the centers for medicare and medicaid services star ratings. *Journal of the American Pharmacists Association*. 2016; 56:e6 |
| Ref ID: 16029 | Ciara Cahill C, Ahern C, Mannix, K. An audit of LDL-c levels post Nurse-led Phase 1 Cardiac Rehabilitation at the University Hospital. *European Journal of Preventive Cardiology*. 2017; 24:S101 |
| Ref ID: 16824 | Burt F, Sirna S, Kashem AM, Bove A. Management of hypercholesterolemia utilizing a home lipid-monitoring system: Preliminary findings∗. *Journal of Clinical Lipidology*. 2012; 6:264-265 |
| Ref ID: 18680 | Andrews S, Marcy T, Osborn B, Planas L. The impact of time my meds medication synchronization program on chronic medication adherence in an adult community pharmacy population. *Journal of the American Pharmacists Association*. 2016; 56:e11 |
| Ref ID: 18845 | Alkhouli MA, Carry BJ, Jarrett H, Sirna SJ. Management of hypercholesterolemia utilizing a home lipid monitoring system: Preliminary findings. *Journal of Clinical Lipidology*. 2013; 7:254-255 |
| Ref ID: 19443 | Pharmacist-managed lipid program reduces medication costs despite increase in drug utilization. Formulary. 2001. 36:378-380 |
| Ref ID: 20464 | Yank V, Agarwal S, Loftus P, Choe C. Use of online crowd-sourcing to identify and study patients with chronic conditions: Is this possible and are findings valid? *Journal of General Internal Medicine*. 2016; 31:S454-S455 |
| Ref ID: 20964 | LePoire A, Wenstrom K, DeVuyst-Miller S, Dettloff R. The impact of patient contact on the rate of primary non-adherence to electronically prescribed medications in a community pharmacy setting. *Journal of the American Pharmacists Association*. 2018; 58:e5-e6 |
| 3 Ref ID: 7423 | Ruiz Bustillo S, Ivern Diaz I, Badosa Marce N, Bruguera Cortada J, Merono Duenas O, Rodriguez Anton D, Perez BA, Fernandez Gasalla A, Marco Navarro E, Comin Colet J. Intensive intervention by specialised nurses after an acute coronary event improves lipid levels and reduces readmissions: a randomized controlled trial. *European heart journal*. 2015; 36:180 |
| 3 Ref ID: 7449 | McDermott MM, Reed G, Greenland P, Mazor K, Pagoto S, Ockene J, Graff R, Merriam PA, Leung K, Manheim L, et al. Telephone counseling to activate peripheral arterial disease patients toward improved low density lipoprotein cholesterol levels: a randomized controlled clinical trial [abstract]. *Circulation: cardiovascular quality and outcomes*. 2011; 4(1):AP268. https://www.ahajournals.org/doi/abs/10.1161/circoutcomes.4.suppl_1.ap268. Accessed November 12, 2019. |
| 3 Ref ID: 7499 | Hillel Yaffe Medical Center. Short Message Service (SMS) Impact on Patient Compliance Receiving Long Term Lipid Lowering Therapy With Statins. Available from: Https://clinicaltrials.gov/show/nct00829348. NLM identifier: NCT00829348. Accessed November 11, 2019. |
| Ref ID: 37898 | Paulós CP, Nygren CE, Celedón C, Cárcamo CA. Impact of a pharmaceutical care program in a community pharmacy on patients with dyslipidemia. *Annals of pharmacotherapy*. 2005; 39:939‐943 |
| Ref ID: 38047 | Pfizer. Compliance With Treatment For Patients With Hyperlipidemia. Available from: https://clinicaltrials.gov/ct2/show/NCT00828945. NLM Identifier: NCT00828945. Accessed November 11, 2019. |
| Ref ID: 38094 | Temple University. Telephone Based Management of Hyperlipidemia. Available from: https://clinicaltrials.gov/ct2/show/NCT01212159. NLM identifier: NCT01212159. Accessed November 11, 2019. |
| **Reason for exclusion:**  **Duplicate** | |
| Ref ID: 7346 | Murimi IB, Chang HY, Daubresse M, Qato DM, Emery SL, Alexander GC. Effect of direct-to-customer advertising (DTCA) on statin use in the United States. *Pharmacoepidemiology and Drug Safety*. 2017; 26:41-42 |
| Ref ID: 12756 | Hae MC, Stevenson JG, Streetman DS, Heisler M, Standiford CJ, Piette JD. Impact of patient financial incentives on participation and outcomes in a statin pill-splitting program. *American Journal of Managed Care*. 2007; 13:298-304 |
| Ref ID: 16370 | Chang HY, Murimi I, Daubresse M, Qato D, Emery S, Alexander GC. Effect of direct-to-consumer advertising (DTCA) on statin use in the United States. *Journal of General Internal Medicine*. 2017; 32:S172 |
| Ref ID: 16371 | Chang H, Murimi IB, Daubresse M, Qato D, Emery SL, Alexander GC. Effect of direct-to-consumer advertising (DTCA) on statin use in the United States. *Value in Health.* 2017; 20:A275 |
| Ref ID: 31433 | Harrison TN, Scott RD, Cheetham TC, Chang SC, Hsu JY, Wei R, Ling Grant DS, Boklage SH, Romo-LeTourneau V, Reynolds K. Trends in Statin Use 2009-2015 in a Large Integrated Health System: Pre- and Post-2013 ACC/AHA Guideline on Treatment of Blood Cholesterol. *Cardiovascular Drugs & Therapy*. 2018; 32:397-404 |
| Ref ID: 33238 | Paulós CP, Akesson Nygren CE, Celedón C, Cárcamo CA. Impact of a pharmaceutical care program in a community pharmacy on patients with dyslipidemia. *Annals of Pharmacotherapy*. 2005; 39:939-943 |
| Ref ID: 34578 | Fabregas M, Berges I, Fina F, Hermosilla E, Coma E, Mendez L, Medina M, Calero S, Serrano E, Morros R, Monteagudo M, Bolibar B. Effectiveness of an intervention designed to optimize statins use: a primary prevention randomized clinical trial. *BMC Family Practice*. 2014; 15:135 |
| Ref ID: 34606 | McAlister FA, Majumdar SR, Padwal RS, Fradette M, Thompson A, Buck B, Dean N, Bakal JA, Tsuyuki R, Grover S, Shuaib A. Case management for blood pressure and lipid level control after minor stroke: PREVENTION randomized controlled trial.[Erratum appears in CMAJ. 2014 Jun 10;186(9):698]. *CMAJ Canadian Medical Association Journal*. 2014; 186:577-84 |
| Ref ID: 35542 | Harats D, Leibovitz E, Maislos M, Wolfovitz E, Chajek-Shaul T, Leitersdorf E, Gavish D, Gerber Y, Goldbourt U, Holem Study Group. Cardiovascular risk assessment and treatment to target low density lipoprotein levels in hospitalized ischemic heart disease patients: results of the HOLEM study. *Israel Medical Association Journal: Imaj*. 2005; 7:355-9 |
| Ref ID: 35577 | Brady AJ, Pittard JB, Grace JF, Robinson PJ. Clinical assessment alone will not benefit patients with coronary heart disease: failure to achieve cholesterol targets in 12,045 patients--the Healthwise II study. *International Journal of Clinical Practice*. 2005; 59:342-5 |
| Ref ID: 35588 | Paulos CP, Nygren CE, Celedon C, Carcamo CA. Impact of a pharmaceutical care program in a community pharmacy on patients with dyslipidemia. *Annals of Pharmacotherapy*. 2005; 39:939-43 |
| Ref ID: 36325 | Robinson JG, Conroy C, Wickemeyer WJ. A novel telephone-based system for management of secondary prevention to a low-density lipoprotein cholesterol < or = 100 mg/dl. *American Journal of Cardiology*. 2000; 85:305-8 |
| Ref ID: 37431 | Shah S, Brown T, Lee JY, Jean-Jacques M, Kandula NR, Persell SD. Individualized risk communication and lay outreach for the primary prevention of cardiovascular disease in community health centers: preliminary results of a randomized controlled trial. *Journal of general internal medicine*. 2014; 29:S126 |
| Ref ID: 37539 | University of Missouri-Columbia. Pilot Program to Improve Statin Adherence and Lower Cholesterol in Older Adults. Available from: Https://clinicaltrials.gov/show/nct01227330. NLM identifier: NCT01227330. Accessed November 12, 2019. |
| Ref ID: 38066 | Lawson Health Research Institute. Randomized Trial Comparing N of 1 Trials to Standard Practice to Improve Adherence to Statins in Patients With Diabetes. Available from: https://clinicaltrials.gov/ct2/show/study/NCT00299169. NLM identifier: NCT00299169. Accessed November 12, 2019. |
| Ref ID: 38156 | Utrecht Institute for Pharmaceutical Sciences. A Community Pharmacist-led Intervention to Improve Adherence to Lipid-lowering Treatment. Available from: https://clinicaltrials.gov/ct2/show/NCT00493337. NLM identifier: NCT00493337. Accessed November 12, 2019. |
| Ref ID: 38364 | AstraZeneca. ORBITAL: Open-Label Primary Care Study: Rosuvastatin Based Compliance Initiatives Linked To Achievement Of LDL Goals. Available from: https://clinicaltrials.gov/ct2/show/NCT00396240. NLM identifier: NCT00396240. Accessed November 12, 2019. |
| **Reason for exclusion:**  **Non-English** | |
| Ref ID: 361 | Yamada H, Nakashima M. A new electronic event monitoring device for recording of medication compliance. *Japanese Journal of Clinical Pharmacology and Therapeutics*. 2001; 32:249-253 |
| Ref ID: 2135 | Tobias Ferrer J, Sanjuán Cortés R, Fàbrega Camprubí M, Bonet Selga L, Roses Circuns C, Boquer Arnó J. Cholesterol is still high. So what do we do now? Treatment of uncontrolled hypercholesterolaemia over a year. *Atencion primaria / Sociedad Española de Medicina de Familia y Comunitaria*. 2002; 29:151-157 |
| Ref ID: 2671 | Svilaas A, Bye P, Strøm EC, Kolbjørnsen O, Tonstad S, Ose L. What happens with patients after participation in a clinical trial? *Tidsskrift for den Norske laegeforening*. 1997; 117:4065-4069 |
| Ref ID: 4414 | Santos RD, Do Nascimento LO, Maranhõo E, Pesquisadores RC. The evaluation of short term effects of awareness programs and pravastatin therapy on subjects from private clinics at high risk for cardiovascular disease. *Arquivos Brasileiros de Cardiologia*. 1997; 69:225-230 |
| Ref ID: 4432 | Santarlasci B, Trippoli S, Bardelli F, Giustini ES, Manfredi C, Messori A. Recommendations for treatment initiations with statins, analysis of prescriptions in Pistoia and assessment of appropriateness by administrative data-base*. Giornale Italiano di Farmacia Clinica*. 2008; 22:459-468 |
| Ref ID: 4731 | Ruiz García A, Villares Rodríguez J, Herreros Tabernero B, Hermosa Hernán J, del Pozo Sosa G, Gordillo López F. Continuing medical education about the use of antilipemic agents in elderly patients aged 65-75 years. *Atencion primaria / Sociedad Española de Medicina de Familia y Comunitaria*. 2001; 27:250-257 |
| Ref ID: 6261 | Pardo Cabello AJ, Bermudo Conde S, Manzano Gamero V, Gómez Jiménez FJ, de la Higuera Torres-Puchol J. Implementation of clinical practice guidelines for acute ischaemic stroke in specialist care centres. *Neurologia*. 2013; 28:137-144 |
| Ref ID: 15153 | Denti L, Annoni V, Campana V, Salvagnini MA, Valenti G. The indication to statin therapy in primary prevention patients with dyslipidemia: implications for using national risk functions in the Italian population. *Italian heart journal*. Supplement : official journal of the Italian Federation of Cardiology. 2004; 5:868-875 |
| Ref ID: 21208 | Sander K, Horn CS, Briesenick C, Sander D. Significant reduction of vascular risk factors after two years of follow-up in the population-based intervention project INVADE. Deutsche Medizinische Wochenschrift. 2006. 131:1853-1859 |
| Ref ID: 34470 | Tarraga Lopez PJ, Garcia-Norro Herreros FJ, Tarraga Marcos L, Solera Albero J, Gonzalez Lopez E, Ruiz Garcia A, Pallares Carratala V, Castro Navarro JL, Alins Presas J, Panisello Royo JM. [Active interventions in hypercholesteroloemia patients with high cardiovascular risk in primary care; estudio ESPROCOL]. *Nutricion Hospitalaria*. 2015; 31:2727-34 |
| Ref ID: 37503 | Márquez E, Casado JJ, López M, Corés E, López JM, Moreno JP, Martín de Pablos JL, Marín J. Therapeutic compliance in cases of lipaemia. Trial on the efficacy of health education. *Atención primaria*. 1998; 22:79‐86 |
| **Reason for exclusion:**  **Interim analysis** | |
| Ref ID: 5499 | Rachmani R, Levi Z, Slavachevski I, Avin M, Ravid M. Teaching patients to monitor their risk factors retards the progression of vascular complications in high-risk patients with Type 2 diabetes mellitus - A randomized prospective study. *Diabetic Medicine*. 2002; 19:385-392 |
| Ref ID: 9371 | Lester WT, Grant R, Barnett GO and Chueh H. Facilitated lipid management using interactive e-mail: preliminary results of a randomized controlled trial. *Medinfo MEDINFO*. 2004;11:232-236. |

Appendix 3. Detailed study demographics

| **Year** | **Author Last Name** | **Location** | **Study Design** | **Sample Size** | **Follow-up Period** | **Outcomes Measured** | **Inclusion Criteria** | **Exclusion Criteria** | **Link to Clinical Trial Registry** | **Included in Meta-Analysis** |
| --- | --- | --- | --- | --- | --- | --- | --- | --- | --- | --- |
| 1996 | Schectman^29^ | United States | RCT | 208 | 24 months | LDL-C, Statin Adherence | Adult veterans; Initial LDL-C >160 mg/dL; two LDL-C tests avg. ≥145 mg/dL with CAD or 2+ cardiac risk factors, or avg. LDL-C ≥175 mg/dL without CAD and <2 cardiac risk factors | TG >250 mg/dL; Thyroid, liver, or kidney abnormality; Severe underlying illness; Diagnosed with DM; Unwilling to stop taking anti-HLD medications 1 month prior to study start |  | ✓ |
| 1997 | Bogden^60^ | United States | RCT | 94 | 6 months | LDL-C | Adults; TC ≥240 mg/dL within 6 months prior to study start | Unable to sign informed consent |  | ✓ |
| 2000 | Nordmann^32^ | Switzerland | RCT | 53 | 6 months | LDL-C, Statin Prescribing | Adults 18-75 years old; Qualifying diagnosis requiring ICU hospitalization; TC >5.5 mmol/L within 24h of hospitalization | Diagnosed with heart failure NYHA III or IV, secondary HCL; severe illness or LEX <1 year; Drugs or conditions affecting lipid metabolism; Substance abuse |  | ✓ |
| 2000 | Nguyen^33^ | France | RCT | 2850 | 1.5 months | LDL-C | Adults 18-75 years old; Treated for HCL with satisfactory response; Appropriate diet | Unwilling to stop taking anti-HLD medications at inclusion |  | ✓ |
| 2000 | Faulkner^66^ | United States | RCT | 30 | 24 months | LDL-C, Statin Adherence | Patients with CABG and/or PTCA 7-30 days prior to inclusion; LDL-C >130 mg/dL; Proficient in English language; Access to a telephone | Serum transaminase >2 x ULN; Concomitant therapy with cyclosporine, warfarin, or erythromycin; Hx of significant GI disease |  | ✓ |
| 2005 | Rachmani^31^ | Israel | RCT | 110 | 96 months | LDL-C, Statin Prescribing | Adults 40-70 years old; Diagnosed with DM Type 2 <10- year duration; BMI <35 kg/m^2^; BP >140/90 mmHg; LDL-C >120 mg/dL; Cr <2 mg/dL; ACR <200 mg/g | Hx of MI, angina pectoris, stroke, any systemic or malignant disease, or previous vascular surgery |  | ✓ |
| 2006 | Lester^36^ | United States | RCT | 235 | 12 months | LDL-C | Adults >30 years old with CAD or CAD risk equivalent; Seen by an eligible PCP within the prior 18 months; LDL-C >100 mg/dL 4-6 months prior to inclusion |  |  | ✓ |
| 2006 | Lee^74^ | United States | RCT | 121 | 14 months | LDL-C | Adults ≥65 years old; Taking 4+ chronic medications daily; Living independently | Assisted living or nursing home residents; Diagnosed with any serious medical condition with LEX <1 year | https://clinicaltrials.gov/ct2/show/NCT00393419 | ✓ |
| 2007 | Khanal^73^ | United States | RCT | 1233 | 24 months | LDL-C, Statin Prescribing | Adults; Diagnosed with angiographic CAD during a diagnostic coronary angiography | Participation in an investigational study in the prior 30 days; Pregnant or breastfeeding; Already being followed by a lipid clinic |  | ✓ |
| 2008 | Riesen^30^ | Switzerland | RCT | 1002 | 6 months | LDL-C, Statin Adherence | Adults; Following PCP for primary HCL; 10-year CHD risk >20%, or diagnosed CHD or other atherosclerotic disease; statin naïve or of an accepted starting dose that was ineffective; TG ≤4.52 mmol/L | Known FH, type III hyperlipoproteinemia, or secondary HCL; Statin intolerant; Pregnant or breastfeeding; Unstable CVD; Uncontrolled DM; Active liver disease; Active liver disease; Cyclosporine therapy; Hx of substance abuse |  | ✓ |
| 2009 | Stacy^26^ | United States | RCT | 497 | 6 months | Statin Adherence | Adults ≥21 years old; Recently filled an index statin prescription with a 30-day supply; Enrolled in HMO or PPO for 12 months prior | Pharmacy claim for lipid-lowering agent in 6 months prior to index statin |  | ✓ |
| 2009 | Willich^85^ | Germany | RCT | 8064 | 12 months | LDL-C, Statin Adherence | Adults; Diagnosed with primary HCL; Statin naïve with LDL-C ≥115 mg/dL or ineffective lipid-lowering treatment with LDL-C ≥125 mg/dL; Hx of CAD or other atherosclerotic disease, 10- year CHD risk ≥20%, or DM | TG >400 mg/dL; FH or secondary HCL; Active liver disease; Creatine kinase > 3 x ULN; Unstable angina |  | ✓ |
| 2009 | McAlister^97^ | Canada | RCT | 637 | 6 months | LDL-C, Statin Prescribing | Adults; Diagnosed with CAD detected by elective cardiac catheterization; Not taking a statin or on an ineffective starting dose; Lipid panel in prior 6 weeks | Statin intolerance; untreated LDL ≤1.8 mmol/L; Emergency CABG; Previously enrolled in ESP-CAD trial; Unknown PCP; Enrolled in other research | https://clinicaltrials.gov/ct2/show/NCT00175240 | ✓ |
| 2010 | Webster^18^ | Australia | RCT | 623 | 2 months | LDL-C | Adults; Residing in Australia; Access to internet |  | https://www.clinicaltrials.gov/ct2/show/NCT00220974 | ✓ |
| 2010 | Villeneuve^99^ | Canada | RCT | 225 | 12 months | LDL-C, Statin Adherence | Adults; Proficient in French or English language; Uses a participating pharmacy in the same cluster as their PCP or had no usual pharmacy; Candidate for or already taking a statin | Prescribed 2+ lipid-lowering medications, Acute CV event in prior 6 months; LEX <1 year; TG >5.9 mmol/L; LDL-C >5.0 mmol/L; Statin intolerance; Participant in clinical trial in prior 2 months | http://www.isrctn.com/ISRCTN66345533 | ✓ |
| 2012 | Nieuwkerk^82^ | Netherlands | RCT | 201 | 18 months | LDL-C, Statin Adherence | Adults; Selected by PCP in outpatient clinics | TC >9.0 mmol/L; TG >4.0 mmol/L; glucose >7.0 mmol/L; Statin medication >3 months prior to inclusion; Hx of substance abuse; Pregnant or breastfeeding; LEX <2 years |  | ✓ |
| 2013 | Zamora^16^ | Spain | RCT | 77 | 3 months | LDL-C | Adults; LDL-C >100 mg/dL; Attending participating centers from January to March 2010; established CVD, 10-year CVD risk ≥5%, and/or DM with other risk factors or target organ damage | Charlson Index >3; LEX <1 year; TG >400 mg/dL |  | ✓ |
| 2013 | Kooy^37^ | Netherlands | RCT | 509 | 12 months | Statin Adherence | Adults >65 years old; Using an eligible community pharmacy; Prescribed statin ≥1 year prior to inclusion with 50 to 80% refill rate | Not personally responsible for medication intake; received medication in a dosing aid; LEX <6 months; Changed statin medication within 540 days prior to inclusion | https://www.clinicaltrials.gov/ct2/show/NCT00493337 | ✓ |
| 2013 | Kardas^38^ | Poland | RCT | 196 | 12 months | Statin Adherence | Adults 40-80 years old; TC ≥250 mg/dL | Mental illness; Assistance in medical care and/or medication intake needed; At risk of not completing study; porphyria, unstable angina, NYHA class III or IV heart failure, or acute liver disease; transaminase ≥3 x ULN; allergy to simvastatin; statin intolerance; Pregnant or lactating |  | ✓ |
| 2013 | Goswami^67^ | United States | RCT | 208 | 6 months | Statin Adherence | Adults >21 years old; Candidate for statin therapy; Met Lipitor physician prescribing information for atorvastatin | Prior atorvastatin prescription; Unwilling to participate in adherence counseling; Unwilling to give informed consent |  | ✓ |
| 2014 | McAlister^79^ | Canada | RCT | 279 | 6 months | LDL-C, Statin Prescribing | Adults; Confirmed ischemic stroke or TIA; LDL-C >2.0 mmol/L; HDL cholesterol ratio >4.0; Avg systolic BP >140 mmHg over 2 visits | Impaired cognition, severe disability or institutionalized; Condition precluding follow-up; Systolic BP ≥200 mmHg; treatment-refractory HTN or dyslipidemia; Enrolled in another clinical trial | https://clinicaltrials.gov/ct2/show/NCT00931788 | ✓ |
| 2014 | Lowrie^96^ | United Kingdom | RCT | 7586 | 12 months | Statin Prescribing | Patients with confirmed ASCVD | Males ≥65 years old diagnosed with HTN | <http://www.isrctn.com/ISRCTN61233866> | ✓ |
| 2015 | Mols^49^ | Denmark | RCT | 189 | 6 months | LDL-C, Statin Adherence | Patients referred for coronary CTA with chest pain; low to intermediate likelihood of CAD; Agatston score ≥70; TC ≥193 mg/dL; LDL-C ≥116 mg/dL | Statin intolerance; Statin use >3 months prior to inclusion; Ongoing participant in cardiac rehab or lifestyle modification program; known CAD, acute coronary syndrome, heart failure, significant heart valve disease; Cognitive dysfunction; Contraindication to coronary CTA |  | ✓ |
| 2015 | Asch^71^ | United States | RCT | 1503 | 12 months | LDL-C | Adults 18 to 80 years old with a consenting primary care provider, 10-year FRS ≥20%, CAD equivalents with an LDL-C ≥120 mg/dL, or FRS 10-20% with LDL-C ≥140 mg/dL | Statin intolerance; Terminal illness making the study protocol unsuitable; Alanine aminotransferase ≥80 U/L, active or progressive liver disease; Unwilling or unable to provide informed consent | https://clinicaltrials.gov/ct2/show/NCT01346189 | ✓ |
| 2015 | Patel^78^ | Australia | RCT | 623 | 36 months | LDL-C | Adults; High CVD risk; Indications for all and no contraindications to any component of at least one of the two polypills | Clinically inappropriate to alter the participant’s medications | ACTRN  12608000  5833347 | ✓ |
| 2016 | Jakobsson^39^ | Sweden | RCT | 201 | 12 months | LDL-C, Statin Prescribing | Patients hospitalized with a diagnosis of MI, unstable angina, stroke, or TIA; Physical and mental capacity to communicate by telephone | Deafness, dementia, aphasia, or other severe disease; Participation in another ongoing trial | http://www.isrctn.com/ISRCTN96595458  http://www.isrctn.com/ISRCTN23868518 | ✓ |
| 2016 | Damush^75^ | United States | RCT | 143 | 6 months | Statin Adherence | Adults; Diagnosed with ischemic stroke or TIA within 12 months prior to inclusion; Proficient in the English language; Access to a telephone; PCP visit within 12 months prior with VA outpatient care; Willing to attend all meetings | LEX <6 months at time of stroke or TIA event; Severe cognitive impairment; Unwilling to participate | https://clinicaltrials.gov/ct2/show/NCT00355147 | ✓ |
| 2018 | Choudhry^72^ | United States | RCT | 2970 | 12 months | LDL-C, Statin Adherence | Adults 18-35 years old; Diagnosed with HLD, HTN, or DM; Nonadherent to medication filled for a qualifying condition; Evidence or poor or worsening disease control; Access to a telephone | <6 months of continuous enrollment in a health plan prior to randomization | https://www.clinicaltrials.gov/ct2/show/NCT02512276 | ✓ |
| 2018 | Mehrpooya^76^ | Iran | RCT | 50 | 6 months | LDL-C | Adults 18-80 years old; Diagnosed with HLD; Indication for statin treatment | Using other anti-HLD medication simultaneously; Hx of psychological disorder; Statin intolerance | https://www.irct.ir/trial/9533 | ✓ |
| 2018 | Martinez^77^ | Spain | RCT | 304 | 24 months | LDL-C | Adults; TC ≥250 mg/dL; Receiving standard care at a participating center; Access to a mobile telephone | Unable to follow-up due to illiteracy, physical disability, severe illness or psychological disorder | https://clinicaltrials.gov/ct2/show/NCT02314663 | ✓ |
| 2018 | Osborn^100^ | United Kingdom | RCT | 184 | 12 months | LDL-C, Statin Prescribing | Adults 30-75 years old; Included on the Quality and Outcomes Framework register for severe mental illness; avg TC ≥5.0 mmol/L, or TC:HDL-C ratio ≥4.0 with 1+ additional CVD risk factors | Under care of acute psychiatric service; Diagnosed with organic psychoses or a personality disorder; LEX <6 months; Pre-existing CVD; Pregnant women | http://www.isrctn.com/ISRCTN13762819 | ✓ |
| 1995 | Shaffer^90^ | United States | Observational | 120 | 18 months | LDL-C | Patients with TC ≥265 mg/dL on two occasions | Patients with acute illness were excluded from LIP; Patients who failed to keep follow-up visits were excluded from GIM |  |  |
| 1996 | Lindholm^34^ | Sweden | RCT | 681 | 18 months | LDL-C | Adults 30-59 years old; moderate HLD and 2+ other CV risk factors; TC ≥6.50 mmol/L on three occasions with the third occasion TC = 6.50-7.79 mmol/L; LDL:HDL ratio ≥4.0 | Secondary HCL; TG ≤4.0 mmol/L; Uncontrolled HTN; Abnormal renal or hepatic function; Raised CPK; Hx of substance abuse; DM Type II; Pancreatitis |  |  |
| 1997 | Shibley^27^ | United States | Observational | 25 | 12 months | LDL-C | Adults ≥20 years old; fasting lipid values above NCEP guidelines; Indication for lipid-lowering; Ability to follow-up | ≤2 months since last hospitalization; Pregnancy within ≤4 months before study start; Initiation of lipid-lowering therapy ≤2 months prior to study start |  |  |
| 1999 | Schwed^28^ | Switzerland | Observational | 39 | 6 months | LDL-C, Statin Adherence | Adult males 20-70 years old; Diagnosed with primary HLD Type II; Willing to cease all lipid-lowering medication for 1 month prior to inclusion | Diagnosed with HLD Type 1, III, IV, or V; MI within 3 months prior to inclusion; severe heart, liver, or kidney failure; insulin-dependent DM; Heavy alcohol consumption; Use of cyclosporin or erythromycin |  |  |
| 2000 | Robinson^88^ | United States | Observational | 2827 | 12 months | LDL-C, Statin Prescribing | Patients at the affiliated hospital with lipid and/or cardiology referrals and patients admitted to the cardiology service |  |  |  |
| 2000 | Birtcher^89^ | United States | Observational |  | 5 months | Statin Prescribing | Patients at the affiliated hospital who were post-AMI and/or PTCA |  |  |  |
| 2001 | Ford^47^ | United Kingdom | Observational | 906 | 12 months | Statin Prescribing | Patients at the affiliated hospital who had a CHD risk request made in 1998 |  |  |  |
| 2002 | Viola^20^ | United States | Observational | 26 | 6 months | LDL-C, Statin Prescribing | Adults; On chronic hemodialysis at the affiliated hospital | Acute renal failure; Pregnant or nursing; Cyclosporine, tacrolimus, or gemfibrozil use; Elevated LAEs; Stain allergy |  |  |
| 2002 | Geber^45^ | United States | Observational | 150 | 24 months | LDL-C | Adults; Seen at VAMC outpatient clinics in the time period; Diagnosed with CAD; LDL-C >100 mg/dL | TSH >5 µIU/mL; HbA_1c_ >8%; TG >400 mg/dL; Terminal illness; Poor short-term LEX; Seen by a private, non-VA provider for HCL |  |  |
| 2002 | Gavish^46^ | Israel | Observational | 1133 | 12 months | LDL-C, Statin Adherence | Patients with ASCVD in need of secondary prevention; Referred by general practice to the lipid clinic |  |  |  |
| 2002 | Hilleman^65^ | United States | Observational | 612 | 26 months | LDL-C; Statin Prescribing | Patients with CHD admitted to the coronary care unit of the affiliated hospital during the specified time period | Hx of HCL; Prior lipid-lowering therapy; Significant renal or hepatic dysfunction |  |  |
| 2003 | Truppo^22^ | United States | Observational | 3077 | 24 months | LDL-C; Statin Adherence | Adults; Noncompliant with statin prescription; continuously enrolled in the health plan during the 12 months prior to implementation | Incomplete prescription records |  |  |
| 2003 | Ryan^94^ | United States | Observational | 417 | 42 months | LDL-C;  Statin Prescribing | High-risk patients diagnosed with dyslipidemia; Referred to the clinic by PCP, cardiologist or self-referral |  |  |  |
| 2003 | Sebregts^95^ | Netherlands | RCT | 184 | 9 months | LDL-C | Adults <70 years old; Admitted to the affiliated hospital during the specified time period; Diagnosed with AMI and/or CABG; Able to participate in regular physiotherapy | Non-Dutch-speaking; Illiterate; Psychiatric disorder that would affect participation |  |  |
| 2004 | Hilleman^40^ | United States | Observational | 612 | 24 months | LDL-C | Patients with CHD; Discharged from the affiliated hospital following an acute coronary event |  |  |  |
| 2004 | de Velasco^51^ | Spain | Observational | 4174 | 6 months | LDL-C, Statin Prescribing | Patients at one of the participating hospitals; Hospitals had to have cardiology service and coronary or ICU, minimum 5 patients weekly with MIs, and agreed to introduce the intervention |  |  |  |
| 2004 | Lappé^64^ | United States | Observational | 57465 | 12 months | Statin Prescribing | Patients at an affiliated hospital; Hospitalized with acute MI, CHD, CHF, or atrial fibrillation | Documented contraindication to the secondary prevention medications |  |  |
| 2005 | Straka^23^ | United States | Nonrandomized Clinical Trial | 481 | 24 months | LDL-C | Adults; Diagnosed with CHD; LDL >105 mg/dL; Followed by a PCP | Terminal illness |  |  |
| 2005 | Harats^42^ | Israel | Observational | 2994 | 2 months | LDL-C | Patients admitted to internal medicine or cardiology wards during the specified time period; Diagnosed with ischemic heart disease | CVA; Acute infection on admission; Active malignant disease; Operation within the 3 months prior to inclusion |  |  |
| 2005 | Bassa^58^ | Spain | Observational | 500 | 12 months | LDL-C | Patients diagnosed with HCL prior to October 1998 |  |  |  |
| 2005 | Brady^87^ | United Kingdom | Observational | 12045 | 6 months | Statin Prescribing | Patients with clinically present CHD |  |  |  |
| 2005 | McLeod^92^ | United Kingdom | Observational | 935 | 60 months | Statin Adherence | Patients diagnosed with CHD; Identified by specialist cardiac rehabilitation nurse during inpatient hospital stay |  |  |  |
| 2005 | Rabinowitz^93^ | Israel | Observational | 150 | 12 months | LDL-C | Adults 30-90 years old attending the clinic; None of the CV risk factors (for the screening group); or, ≥1 CV risk factors (for the monitoring group) | Active malignant disease |  |  |
| 2005 | Paulós^101^ | Chile | RCT | 42 | 4 months | LDL-C, Statin Adherence | Adults; Customer of the affiliated pharmacy; Diagnosed with DLP; Currently treated for DLP | Communication difficulties; Pregnancy; Self-medication |  |  |
| 2006 | Vrijens^19^ | Belgium | RCT | 392 | 12 months | Statin Adherence | Adults; Taking atorvastatin for ≥3 months prior to inclusion | Contraindication to continuation of atorvastatin treatment |  |  |
| 2006 | de Lusignan^52^ | United Kingdom | Observational | 29915 | 8 months | Statin Prescribing | Patients at one of the 99 participating practices; Diagnosed with ≥1 CV diseases |  |  |  |
| 2006 | Rehring^61^ | United States | Observational | 691 | 6 months | LDL-C | Patient at the affiliated health care system; Diagnosed with PAD; ABI <0.9 | Diagnosed with CAD |  |  |
| 2007 | Goldberg^43^ | United States | Observational | 4048 | 27 months | LDL-C | Patients of the affiliated primary care clinic; Active prescription for simvastatin; Received ≥1 refill of simvastatin following initial prescription; LDL-C target on 100 mg/dL | Prescribed the recommended maximum daily dose or higher of simvastatin |  |  |
| 2007 | Choe^91^ | United States | RCT | 111 | 6 months | LDL-C, Statin Adherence | Patients diagnosed with HLD; ≥1 prescription for atorvastatin, simvastatin, or pravastatin over a 6-month period; Candidate for pill splitting; Approval from PCP | Changed health center or prescription benefit plan; No longer taking one of the specified medications; Deceased |  |  |
| 2008 | Stockl^24^ | United States | Observational | 1144 | 4 months | Statin Prescribing, Statin Adherence | Members of the MA-PD and PDP; Met the 3 MTMP criteria; Diagnosed with DM or CAD No pharmacy claims data for a statin during identification period | Previously declined to participate in MTMP |  |  |
| 2008 | Hatfield^62^ | United Kingdom | Observational | 78 | 3 months | LDL-C, Statin Adherence | Patients with newly diagnosed intermittent claudication; Referred from an outpatient clinic | Coexisting CHD |  |  |
| 2008 | Coodley^84^ | United States | Observational | 1375 | 8 months | LDL-C | Patients attending the affiliated clinic during the specified timeframe; Had results from a fasting lipid panel; Willing to consider lipid-lowering therapy | Determined to be low risk or controlled; Statin intolerance; Extreme comorbidity; TG >400 mg/dL |  |  |
| 2008 | Hung^86^ | Taiwan | RCT | 200 | 6 months | LDL-C, Statin Prescribing | Patients with angiographically-proven CHD of at least 50% stenosis | Receiving lipid-lowering therapy; Involvements in non-coronary studies; Lost to follow-up; Deceased or bedridden |  |  |
| 2009 | Stephenson^25^ | United States | Observational | 681 | 12 months | LDL-C | Patients diagnosed with FH; Registered with MEDPED | Already having access to a lipid specialist; Met NCEP ATPIII goal |  |  |
| 2009 | Lima^35^ | Brazil | Observational | 87 | 6 months | LDL-C | Adults >30 years old; Referred for suspicion of elevated CV risk; HIV infection on HAART; FRS >10%, or ATP III risk categories CHD and CHD risk equivalent, or multiple risk factors | Enrolled in a CV risk prevention program; Previous coronary angioplasty, bypass surgery, MI, or CVA; Cr >2.0 mg/dL; Diagnosed with FH or serious illness; Use of anti-HLD drugs; Active infection; Alcoholism |  |  |
| 2009 | Casebeer^54^ | United States | Observational | 1949 | 4 months | Statin Adherence | Adults ≥40 years old; Received a new lipid-lowering prescription in the 30 days prior; Proficient in the English language; Provided written consent to receive mailings | Previous use of a cholesterol-lowering medication in the 12 months prior |  |  |
| 2010 | Bhattacharyya^57^ | Canada | RCT | 340 | 10 months | LDL-C, Statin Prescribing | Adults residing in one of the studied communities; Diagnosed with diabetes; On the diabetes registry | Pregnant women |  |  |
| 2010 | Chen^70^ | Taiwan | Observational | 200 | 12 months | LDL-C | Patients attending the affiliated clinic for >1 year prior to their current visit; |  |  |  |
| 2011 | Gitt^44^ | Germany | Observational | 3131 | 9 months | LDL-C | Patients diagnosed with CHD and/or DM and/or PAD, or a 10-year CHD risk >20%; Prescribed chronic statin treatment |  |  |  |
| 2011 | Chung^53^ | Hong Kong | Observational | 300 | 24 months | LDL-C | Adults diagnosed with dyslipidemia; On primary or secondary prevention for CHD | Pregnant women; Unable to give informed consent |  |  |
| 2011 | Schmittdiel^83^ | United States | Observational | 100298 | 15 months | LDL-C | Adults with a new statin prescription which was refilled ≥1 time; Active pharmacy benefit for 2 years prior to index date; LDL-C result within the 12 months prior and 3-15 months post index date |  |  |  |
| 2012 | Aziz^63^ | United States | Observational | 484 | 12 months | LDL-C, Statin Prescribing | Patients admitted for acute coronary syndromes from the Advanced Cardiac Admission Program |  |  |  |
| 2012 | Farley^69^ | United States | Observational | 74748 | 24 months | Statin Adherence | Adults continuously enrolled in the affiliated health plan during the specified time period; Taking ≥1 of the studied classes of drugs | Change in health plan enrollment status during the study |  |  |
| 2013 | Dresser^50^ | Canada | RCT | 1272 | 6 months | LDL-C | Adults; Diagnosed with HTN and DLP; ≥1 uncontrolled risk factor | Required 3+ medications to control HTN or 2+ to control DLP; Diagnosed with ischemic heart disease; AFib, PAD, CVA, or chronic kidney disease; active participation in other HTN/DLP studies | http://clini  caltrials.gov/ct2/show/NCT00637078 |  |
| 2013 | Brath^55^ | Austria | RCT | 53 | 13 months | LDL-C, Statin Adherence | Patients at the affiliated clinic with ≥2 of the following: DM Type II, HTN, HCL |  |  |  |
| 2013 | Derose^81^ | United States | RCT | 5216 | 2.5 months | Statin Adherence | Adults ≥24 years old; Nonadherent for a new statin prescription; Member of the health plan for ≥1 year | Statin dispensed or health plan enrollment gap of >30 days within 12 months prior to index date |  |  |
| 2014 | Clark^68^ | United States | Observational | 4596 | 18 months | Statin Adherence | Active health plan participants; Diagnosed with CAD, HLD, and/or DM; Eligible for ≥1 of the programs; ≥1 medication claim for treatment of DM or HLD |  |  |  |
| 2014 | Shoulders^80^ | United States | Observational | 126 | 12 months | LDL-C, Statin Prescribing | Adults; Prescribed a medication containing simvastatin that required dosage adjustment | Discontinuation of the simvastatin-containing medication prior to the specified date |  |  |
| 2015 | Vinker^21^ | Israel | Observational | 433662 | 18 months | LDL-C, Statin Prescribing | Patients of the affiliated health system who remained in the same risk group throughout the study period |  |  |  |
| 2015 | Persell^98^ | United States | RCT | 646 | 12 months | LDL-C, Statin Prescribing | Men ≥35 and women ≥45 years old; LDL-C ≥100mg/dL in the most recent result in the past 5 years; Not actively taking lipid-lowering medication; ≥1 visit with a study site within the 6 months prior to randomization; 10-year FRS score ≥10% | Diagnosed with CAD, PAD, carotid artery disease, abdominal aortic aneurysm, or DM; Primary language other than English or Spanish | https://clinicaltrials.gov/ct2/show/NCT01610609 |  |
| 2016 | Harrison^41^ | United States | Observational | 49686 | 12 months | LDL-C, Statin Adherence | Adults on the system’s HTN and CVD registries; Statin or lisinopril-HCTZ prescription 2-6 weeks overdue; Member of a prescription benefit plan | Declined to be contacted; Allergy or intolerance to the medication; Pregnant women |  |  |
| 2017 | Bosworth^56^ | United States | RCT | 240 | 12 months | LDL-C, Statin Adherence | Veterans; Enrolled in PCP clinic for ≥1 year; PCP visit in the past year; Diagnosed with HCL; LDL-C >130 mg/dL and/or <80% statin adherence in the last 12 months; Prescribed a qualifying dose of a statin medication |  | https://clinicaltrials.gov/ct2/show/results/NCT01744977 |  |
| 2017 | Andrews^59^ | United States | Observational | 380 | 6 months | Statin Adherence | Adults; ≥1 oral diabetes, renin-angiotension system antiagonist, or statin medication on prescription list within the past 6 months; Enrolled in the usual care program at an eligible pharmacy |  |  |  |
| 2018 | Weng^17^ | United Kingdom | Observational | 118 | 6 months | LDL-C, Statin Prescribing | Adults; TC > 7.5 mmol/L documented in EHR record from one of the affiliated practices | Confirmed diagnosis of FH |  |  |
| 2018 | Etxeberria^48^ | Spain | RCT | 137543 | 18 months | Statin Prescribing | Patients ≥14 years old; Visit with PCP during baseline and post-intervention; Diagnosed with DM Type 2 or HTN, or eligible for coronary risk assessment, or started a statin or diagnosed with CHD in baseline or post-intervention period | Patients assigned to a non-participating PCP; Health units that declined to participate | http://www.isrctn.com/ISRCTN88876909 |  |

Appendix 4. Count of implementation strategy organized by category and strategy

|  | Use Evaluative and Iterative Strategies | | | | | | | | Adapt and Tailor to the Context | | | Develop Stakeholder Relationships | | | | | | | | Train and educate the stakeholders | | | | | | Support Clinicians | | | | Engage Consumers | | | Utilize financial strategies | | Change Infrastructure | | | |  |
| --- | --- | --- | --- | --- | --- | --- | --- | --- | --- | --- | --- | --- | --- | --- | --- | --- | --- | --- | --- | --- | --- | --- | --- | --- | --- | --- | --- | --- | --- | --- | --- | --- | --- | --- | --- | --- | --- | --- | --- |
| **Implementation strategy** | Assess Readiness and Identify Barriers and Facilitators | Audit and Provide Feedback | Conduct Local Needs Assessment | Develop and Implement Tools for Quality Monitoring | Develop and Organize Quality Monitoring Systems | Obtain and Use Patient/Consumer and Family Feedback | Purposely Reexamine the Implementation | Stage Implementation Scale Up | Promote Adaptability | Tailor Strategies | Use Data Experts | Build A Coalition | Conduct Local Consensus Discussions | Identify and Prepare Champions | Inform Local Opinion Leaders | Involve Executive Boards | Obtain Formal Commitments | Organize Clinician Implementation Team Meetings | Use Advisory Boards and Workgroups | Conduct Educational Meetings | Conduct Educational Outreach Visits | Develop Educational Materials | Distribute Educational Materials | Provide Ongoing Consultation | Work with Education Institutions | Create New Clinical Teams | Facilitate Relay of Clinical Data to Providers | Remind Clinicians | Revise Professional Roles | Intervene with Patients/Consumers to Enhance Uptake and Adherence | Involve Patients/Consumers and Family Members | Prepare Patient/Consumers to Be Active Participants | Alter Incentive/Allowance Structures | Alter Patient/Consumer Fees | Change Physical Structure and Equipment | Change Record Systems | Create or Change Credentialing And/Or Licensure Standards | Mandate Change | **n** |
| **n** | 1 | 6 | 2 | 6 | 12 | 2 | 1 | 3 | 2 | 1 | 1 | 1 | 2 | 2 | 2 | 1 | 1 | 1 | 1 | 11 | 15 | 11 | 41 | 1 | 1 | 18 | 23 | 20 | 7 | 41 | 2 | 4 | 1 | 5 | 1 | 6 | 1 | 1 |  |
| Zamora, 2013 |  |  |  |  |  |  |  |  |  |  |  |  |  |  |  |  |  |  |  |  |  |  |  |  |  |  | X |  |  |  |  |  |  |  |  |  |  |  | 1 |
| Weng, 2018 |  |  |  | X |  |  |  |  |  |  |  |  |  |  |  |  |  |  |  |  | X |  |  |  |  |  | X | X |  |  |  |  |  |  |  |  |  |  | 4 |
| Webster, 2010 |  |  |  |  | X |  |  |  |  |  |  |  |  |  |  |  |  |  |  |  |  |  | X |  |  |  |  |  |  |  |  |  |  |  |  |  |  |  | 2 |
| Vrijens, 2006 |  |  |  |  |  |  |  |  |  |  |  |  |  |  |  |  |  |  |  |  | X |  | X |  |  |  |  |  |  | X |  |  |  |  |  |  |  |  | 3 |
| Viola, 2002 |  |  |  |  | X |  |  |  |  |  |  |  |  |  |  |  |  |  |  |  |  | X | X |  |  | X | X |  |  |  |  |  |  |  |  |  | X |  | 6 |
| Vinker, 2015 |  |  |  |  | X |  |  |  |  |  |  |  |  |  |  |  |  |  |  |  |  |  |  |  |  |  | X | X |  |  |  |  |  |  |  |  |  |  | 3 |
| Truppo, 2003 |  |  |  |  |  | X |  |  |  |  |  |  |  |  |  |  |  |  |  |  |  |  | X |  |  |  | X |  |  | X |  |  |  |  |  |  |  |  | 4 |
| Straka, 2005 |  |  |  |  |  |  |  |  |  |  |  |  |  |  |  |  |  |  |  |  |  |  |  |  |  | X | X |  |  | X |  |  |  |  |  |  |  |  | 3 |
| Stockl, 2008 |  |  |  |  |  |  |  |  |  |  |  |  |  |  |  |  |  |  |  |  |  |  | X |  |  |  | X |  |  |  |  |  |  |  |  |  |  |  | 2 |
| Stephenson, 2009 |  |  |  |  |  |  |  |  |  |  |  |  |  |  |  |  |  |  |  |  |  |  | X |  |  |  |  |  |  | X |  |  |  |  |  |  |  |  | 2 |
| Stacy, 2009 |  |  |  |  |  |  |  |  |  |  |  |  |  |  |  |  |  |  |  |  |  |  | X |  |  |  |  |  |  | X |  |  |  |  |  |  |  |  | 2 |
| Shibley, 1997 |  |  |  |  |  |  |  |  |  |  |  |  |  |  |  |  |  |  |  |  | X |  | X |  |  | X | X |  |  |  |  |  |  |  |  |  |  |  | 4 |
| Schwed, 1999 |  |  |  |  |  |  |  |  |  |  |  |  |  |  |  |  |  |  |  |  |  |  |  |  |  |  |  |  |  | X |  |  |  |  |  |  |  |  | 1 |
| Schectman, 1996 |  |  |  |  |  |  |  |  |  |  |  |  |  |  |  |  |  |  |  |  | X |  |  |  |  | X |  |  |  | X |  |  |  |  |  |  |  |  | 3 |
| Riesen, 2008 |  |  |  |  |  |  |  |  |  |  |  |  |  |  |  |  |  |  |  |  |  |  | X |  |  |  |  |  |  | X |  |  |  |  |  |  |  |  | 2 |
| Rachmani, 2005 |  |  |  |  |  |  |  |  |  |  |  |  |  |  |  |  |  |  |  |  |  |  | X |  |  |  |  | X |  | X |  |  |  |  |  |  |  |  | 3 |
| Nordmann, 2000 |  |  |  |  |  |  |  |  |  |  |  |  |  |  |  |  |  |  |  |  |  |  | X |  |  |  | X |  |  |  |  |  |  |  |  |  |  |  | 2 |
| Nguyen, 2000 |  |  |  |  |  |  |  |  |  |  |  |  |  |  |  |  |  |  |  |  | X | X | X |  |  |  |  |  |  |  |  |  |  |  |  |  |  |  | 3 |
| Lindholm, 1996 |  |  |  |  |  |  |  |  |  |  |  |  |  |  |  |  |  |  |  |  | X |  | X |  |  |  |  |  |  |  |  |  |  |  |  |  |  |  | 2 |
| Lima, 2009 |  |  |  |  |  |  |  |  |  |  |  |  |  |  |  |  |  |  |  |  |  |  |  |  |  |  |  |  |  |  |  | X |  |  |  |  |  |  | 1 |
| Lester, 2006 |  |  |  |  |  |  |  |  |  |  |  |  |  |  |  |  |  |  |  |  |  |  |  |  |  |  | X | X |  |  |  |  |  |  |  | X |  |  | 3 |
| Kooy, 2013 |  |  |  |  |  |  |  |  |  |  |  |  |  |  |  |  |  |  |  |  |  |  |  |  |  |  |  |  |  | X |  |  |  |  |  |  |  |  | 1 |
| Kardas, 2013 |  |  |  |  |  |  |  |  |  |  |  |  |  |  |  |  |  |  |  |  |  |  | X |  |  |  |  |  |  | X |  |  |  |  |  |  |  |  | 2 |
| Jakobsson, 2016 |  |  |  |  |  |  |  |  |  |  |  |  |  |  |  |  |  |  |  |  |  |  | X |  |  |  |  |  |  |  |  |  |  |  |  |  |  |  | 1 |
| Hilleman, 2004 |  |  |  |  |  |  |  |  |  |  |  |  |  |  |  |  |  |  |  |  |  |  |  |  |  |  |  | X | X |  |  |  |  |  |  |  |  |  | 2 |
| Harrison, 2016 |  |  |  |  | X |  |  |  |  |  |  |  |  |  |  |  |  |  |  |  |  |  |  |  |  |  |  |  |  | X |  |  |  |  |  |  |  |  | 2 |
| Harats, 2005 |  |  |  |  |  |  |  |  |  |  |  |  |  |  |  |  |  |  |  | X |  |  |  |  |  |  |  |  |  |  |  |  |  |  |  |  |  |  | 1 |
| Goldberg, 2007 |  |  |  |  |  |  |  |  |  |  |  |  |  |  |  |  |  |  |  | X | X | X | X |  |  |  |  |  |  |  |  |  |  |  |  |  |  |  | 4 |
| Gitt, 2011 |  |  |  |  |  |  |  |  |  |  |  |  |  |  |  |  |  |  |  |  |  |  |  |  |  |  | X |  |  |  |  |  |  |  |  |  |  |  | 1 |
| Geber, 2002 |  |  |  |  |  |  |  |  |  |  |  |  |  |  |  |  |  |  |  |  |  |  |  |  |  |  |  | X |  |  |  |  |  |  |  |  |  |  | 1 |
| Gavish, 2002 |  |  |  |  |  |  |  |  |  |  |  |  |  |  |  |  |  |  |  |  |  |  |  |  |  |  |  |  |  | X |  |  |  |  |  |  |  |  | 1 |
| Ford, 2001 |  |  |  |  |  |  |  |  |  |  |  |  |  |  |  |  |  |  |  |  |  |  |  |  |  |  | X |  |  |  |  |  |  |  |  |  |  |  | 1 |
| Etxeberria, 2018 |  |  |  |  |  |  |  |  |  |  |  |  |  |  |  |  |  |  |  | X | X |  | X |  |  |  |  |  |  |  |  |  |  |  |  |  |  |  | 3 |
| Mols, 2015 |  |  |  |  |  |  |  |  |  |  |  |  |  |  |  |  |  |  |  |  |  |  |  |  |  |  |  |  |  | X |  |  |  |  |  |  |  |  | 1 |
| Dresser, 2013 |  |  |  |  |  |  |  |  |  |  |  |  |  |  |  |  |  |  |  |  | X | X | X | X |  |  |  |  |  |  |  |  |  |  |  |  |  |  | 4 |
| de Velasco, 2004 |  |  |  |  |  |  |  |  |  |  |  |  |  |  |  |  |  |  |  | X |  | X | X |  |  |  |  |  |  |  | X |  |  |  |  |  |  |  | 4 |
| de Lusignan, 2006 |  | X |  | X |  |  |  | X |  |  |  |  |  |  |  |  |  |  |  |  |  |  |  |  |  |  |  |  |  |  |  |  |  |  |  |  |  |  | 3 |
| Chung, 2011 |  |  |  |  |  |  |  |  |  |  |  |  |  |  |  |  |  |  |  |  |  |  | X |  |  | X |  |  |  | X |  |  |  |  |  |  |  |  | 3 |
| Casebeer, 2009 |  |  |  |  |  |  |  |  |  |  |  |  |  |  |  |  |  |  |  |  |  |  | X^*^ |  |  |  |  |  |  |  |  |  |  |  |  |  |  |  | 1 |
| Brath, 2013 |  |  |  |  |  |  |  |  |  |  |  |  |  |  |  |  |  |  |  |  |  |  |  |  |  |  |  |  |  | X |  |  |  |  |  | X |  |  | 2 |
| Bosworth, 2017 |  |  |  |  |  |  |  |  |  |  |  |  |  |  |  |  |  |  |  |  |  |  |  |  |  |  |  |  |  | X |  |  |  |  |  |  |  |  | 1 |
| Bhattacharyya, 2010 |  | X |  |  |  |  |  |  |  |  |  |  |  |  |  |  |  |  |  |  | X |  |  |  |  |  |  |  |  |  |  |  |  |  |  |  |  |  | 2 |
| Bassa, 2005 |  |  |  | X | X |  |  |  |  |  |  |  |  |  |  |  | X |  |  |  |  |  |  |  |  |  | X | X |  |  |  |  |  |  |  | X |  |  | 6 |
| Andrews, 2017 |  |  |  |  | X |  |  |  |  |  |  |  |  |  |  |  |  |  |  |  | X |  |  |  |  |  |  |  |  | X |  |  |  |  |  |  |  |  | 3 |
| Bogden, 1997 |  |  |  |  |  |  |  |  |  |  |  |  |  |  |  |  |  |  |  |  |  |  |  |  |  | X |  |  | X |  |  | X |  |  |  |  |  |  | 3 |
| Rehring, 2006 |  |  |  |  | X |  |  | X |  |  |  |  |  |  |  |  |  |  |  |  |  |  |  |  |  | X | X |  |  |  |  |  |  |  |  |  |  |  | 4 |
| Hatfield, 2008 |  |  |  |  |  |  |  |  |  |  |  |  |  |  |  |  |  |  |  |  | X |  |  |  |  | X |  |  |  |  |  |  |  |  |  |  |  |  | 2 |
| Aziz, 2012 |  | X |  | X | X |  | X |  | X |  | X |  |  | X |  |  |  | X |  |  |  | X | X |  |  | X |  | X |  |  |  |  |  |  |  |  |  | X | 13 |
| Lappé, 2004 |  | X |  | X | X |  |  |  |  |  |  |  |  |  |  |  |  |  |  | X |  | X | X |  |  |  |  | X |  |  |  |  |  |  |  | X |  |  | 8 |
| Hilleman, 2002 |  |  |  |  |  |  |  |  |  |  |  |  |  |  |  |  |  |  |  |  |  |  |  |  |  |  |  | X |  |  |  |  |  |  |  |  |  |  | 1 |
| Faulkner, 2000 |  |  |  |  |  |  |  |  |  |  |  |  |  |  |  |  |  |  |  |  |  |  |  |  |  |  |  |  |  | X |  |  |  |  |  |  |  |  | 1 |
| Goswami, 2013 |  |  |  |  |  |  |  |  |  |  |  |  |  |  |  |  |  |  |  |  |  |  | X |  |  |  |  |  |  | X |  |  |  | X |  |  |  |  | 3 |
| Clark, 2014 |  |  |  |  |  |  |  |  |  |  |  |  |  |  |  |  |  |  |  |  |  |  |  |  |  |  |  |  |  |  |  |  |  | X |  |  |  |  | 1 |
| Farley, 2012 |  |  |  |  |  |  |  |  |  |  |  |  |  |  |  |  |  |  |  |  |  |  |  |  |  |  |  |  |  |  |  |  |  | X |  |  |  |  | 1 |
| Chen, 2010 |  |  |  |  |  |  |  |  |  |  |  |  |  |  |  |  |  |  |  |  |  |  |  |  |  |  | X | X |  |  |  |  |  |  |  | X |  |  | 3 |
| Asch, 2015 |  |  |  | X |  |  |  |  |  |  |  |  |  |  |  |  |  |  |  |  |  |  |  |  |  |  |  |  |  | X |  |  | X |  |  |  |  |  | 3 |
| Choudhry, 2018 |  |  |  |  | X |  |  |  |  |  |  |  |  |  |  |  |  |  |  | X |  | X | X |  |  |  | X | X |  | X |  |  |  |  |  | X |  |  | 8 |
| Khanal, 2007 |  |  |  |  |  |  |  |  |  |  |  |  |  |  |  |  |  |  |  |  |  |  |  |  |  | X |  |  | X |  |  |  |  |  |  |  |  |  | 2 |
| Lee, 2006 |  |  |  |  |  |  |  |  |  |  |  |  |  |  |  |  |  |  |  |  |  |  | X |  |  | X |  |  |  | X |  |  |  |  |  |  |  |  | 3 |
| Damush, 2016 |  |  |  |  |  |  |  |  |  | X |  |  |  |  |  |  |  |  |  |  |  |  | X |  |  |  |  |  |  | X |  |  |  |  |  |  |  |  | 3 |
| Mehrpooya, 2018 |  |  |  |  |  |  |  |  |  |  |  |  |  |  |  |  |  |  |  |  |  |  |  |  |  |  |  |  |  | X |  |  |  |  |  |  |  |  | 1 |
| Martinez, 2018 |  |  |  |  |  |  |  |  |  |  |  |  |  |  |  |  |  |  |  |  |  |  | X |  |  |  |  |  |  | X |  |  |  |  |  |  |  |  | 2 |
| Patel, 2015 |  |  |  |  |  |  |  |  |  |  |  |  |  |  |  |  |  |  |  |  |  |  |  |  |  |  |  |  |  | X |  |  |  |  |  |  |  |  | 1 |
| McAlister, 2014 |  |  |  |  |  |  |  |  |  |  |  |  |  |  |  |  |  |  |  |  |  |  | X |  |  | X | X |  |  | X |  |  |  |  |  |  |  |  | 4 |
| Shoulders, 2014 |  |  |  |  |  |  |  |  |  |  |  |  |  |  |  |  |  |  |  |  | X |  |  |  |  |  | X |  |  |  |  |  |  |  |  |  |  |  | 2 |
| Derose, 2013 |  |  |  |  |  |  |  |  |  |  |  |  |  |  |  |  |  |  |  |  |  |  | X |  |  |  |  |  |  | X |  |  |  |  |  |  |  |  | 2 |
| Nieuwkerk, 2012 |  |  |  |  |  |  |  |  |  |  |  |  |  |  |  |  |  |  |  |  |  |  | X |  |  |  |  |  |  | X |  |  |  |  |  |  |  |  | 2 |
| Schmittdiel, 2011 |  |  |  |  |  |  |  |  |  |  |  |  |  |  |  |  |  |  |  |  |  |  |  |  |  |  |  |  |  | X |  |  |  |  |  |  |  |  | 1 |
| Coodley, 2008 |  |  |  |  |  |  |  |  |  |  |  |  |  |  |  |  |  |  |  |  |  |  |  |  |  |  | X | X |  | X |  |  |  |  |  |  |  |  | 3 |
| Willich, 2009 |  |  |  |  |  |  |  |  |  |  |  |  |  |  |  |  |  |  |  |  |  |  |  |  |  |  |  |  |  | X |  |  |  |  |  |  |  |  | 1 |
| Hung, 2008 |  |  |  |  |  |  |  |  |  |  |  |  |  |  |  |  |  |  |  |  |  |  |  |  |  |  |  | X |  |  |  |  |  | X |  |  |  |  | 2 |
| Brady, 2005 |  | X |  |  | X |  |  |  |  |  |  |  |  |  |  |  |  |  |  |  |  |  | X |  |  |  | X | X |  |  |  |  |  |  |  |  |  |  | 5 |
| Robinson, 2000 |  |  |  |  |  |  |  |  |  |  |  |  |  |  |  |  |  |  |  |  |  |  | X |  |  | X |  |  |  | X |  |  |  |  |  |  |  |  | 3 |
| Birtcher, 2000 |  |  | X |  |  |  |  |  |  |  |  |  | X | X |  | X |  |  |  | X |  |  |  |  |  |  |  | X | X |  |  |  |  |  |  |  |  |  | 7 |
| Shaffer, 1995 |  |  |  |  |  |  |  |  |  |  |  |  |  |  |  |  |  |  |  |  |  |  | X |  |  | X |  |  |  | X |  |  |  |  |  |  |  |  | 3 |
| Choe, 2007 |  |  |  |  |  |  |  |  |  |  |  |  |  |  |  |  |  |  |  |  |  |  | X |  |  |  |  | X |  | X |  |  |  | X |  |  |  |  | 4 |
| McLeod, 2005 |  |  |  |  |  |  |  |  |  |  |  |  |  |  |  |  |  |  |  |  |  |  |  |  |  | X |  |  |  |  |  |  |  |  |  |  |  |  | 1 |
| Rabinowitz, 2005 |  |  |  |  |  |  |  |  |  |  |  |  |  |  |  |  |  |  |  |  |  |  | X |  |  | X | X | X | X |  |  |  |  |  |  |  |  |  | 5 |
| Ryan, 2003 |  |  |  |  |  |  |  |  |  |  |  |  |  |  |  |  |  |  |  |  |  |  |  |  |  | X |  |  |  | X |  |  |  |  |  |  |  |  | 2 |
| Sebregts, 2003 |  |  |  |  |  |  |  |  |  |  |  |  |  |  |  |  |  |  |  |  |  |  | X |  |  |  |  |  |  |  |  | X |  |  |  |  |  |  | 2 |
| Lowrie, 2014 | X | X | X |  | X |  |  |  |  |  |  |  |  |  | X |  |  |  |  | X | X |  |  |  |  |  |  | X | X |  |  |  |  |  |  |  |  |  | 9 |
| McAlister, 2009 |  |  |  |  |  |  |  |  |  |  |  |  |  |  | X |  |  |  |  |  |  |  |  |  |  |  |  | X |  |  |  |  |  |  |  |  |  |  | 2 |
| Persell, 2015 |  |  |  |  |  |  |  |  |  |  |  |  |  |  |  |  |  |  |  | X |  | X | X |  | X |  | X |  | X | X |  |  |  |  |  |  |  |  | 7 |
| Villeneuve, 2010 |  |  |  |  |  |  |  |  |  |  |  | X |  |  |  |  |  |  |  |  | X |  |  |  |  | X | X |  |  | X |  | X |  |  |  |  |  |  | 6 |
| Osborn, 2018 |  |  |  |  |  | X |  | X | X |  |  |  | X |  |  |  |  |  | X | X |  | X | X |  |  |  |  |  |  | X | X |  |  |  |  |  |  |  | 10 |
| Paulos, 2005 |  |  |  |  |  |  |  |  |  |  |  |  |  |  |  |  |  |  |  | X |  | X | X |  |  |  |  |  |  | X |  |  |  |  | X |  |  |  | 5 |

Appendix 5. Detailed Proctor’s framework description of each strategy

| **AUTHOR, YEAR** | **IMPLEMENTATION STRATEGY CATEGORY** | **ACTOR** | **ACTION** | **ACTION TARGET** | **TEMPORALITY** | **DOSE** | **IMPLEMENTATION OUTCOME AFFECTED** | **JUSTIFICATION** |
| --- | --- | --- | --- | --- | --- | --- | --- | --- |
| Zamora, 2013 | FACILITATE RELAY OF CLINICAL DATA TO PROVIDERS | X | X | X |  |  |  |  |
| Weng, 2018 | CONDUCT EDUCATIONAL OUTREACH VISITS | X | X | X | X | X |  |  |
| Weng, 2018 | DEVELOP AND IMPLEMENT TOOLS FOR QUALITY MONITORING | X | X | X | X | X |  |  |
| Weng, 2018 | FACILITATE RELAY OF CLINICAL DATA TO PROVIDERS | X | X | X |  | X |  |  |
| Weng, 2018 | REMIND CLINICIANS | X | X | X | X |  |  |  |
| Webster, 2010 | DISTRIBUTE EDUCATIONAL MATERIALS | X | X | X | X |  |  |  |
| Webster, 2010 | DEVELOP AND ORGANIZE QUALITY MONITORING SYSTEMS | X | X | X | X |  |  |  |
| Vrijens, 2006 | CONDUCT EDUCATIONAL OUTREACH VISITS | X | X | X |  |  |  |  |
| Vrijens, 2006 | DISTRIBUTE EDUCATIONAL MATERIALS | X | X | X | X | X |  |  |
| Vrijens, 2006 | INTERVENE WITH PATIENTS/CONSUMERS TO ENHANCE UPTAKE AND ADHERENCE | X | X | X | X | X |  |  |
| Viola, 2002 | CREATE NEW CLINICAL TEAMS | X | X | X |  |  |  |  |
| Viola, 2002 | CREATE OR CHANGE CREDENTIALING AND/OR LICENSURE STANDARDS | X | X | X |  |  |  |  |
| Viola, 2002 | DEVELOP AND ORGANIZE QUALITY MONITORING SYSTEMS | X | X | X | X | X |  |  |
| Viola, 2002 | DEVELOP EDUCATIONAL MATERIALS | X | X | X | X | X |  |  |
| Viola, 2002 | DISTRIBUTE EDUCATIONAL MATERIALS | X | X | X | X | X |  |  |
| Viola, 2002 | FACILITATE RELAY OF CLINICAL DATA TO PROVIDERS | X | X | X | X | X |  |  |
| Vinker, 2015 | DEVELOP AND ORGANIZE QUALITY MONITORING SYSTEMS | X | X | X | X | X |  |  |
| Vinker, 2015 | FACILITATE RELAY OF CLINICAL DATA TO PROVIDERS | X | X | X | X | X |  |  |
| Vinker, 2015 | REMIND CLINICIANS | X | X | X | X | X |  |  |
| Truppo, 2003 | DISTRIBUTE EDUCATIONAL MATERIALS | X | X | X | X | X |  |  |
| Truppo, 2003 | FACILITATE RELAY OF CLINICAL DATA TO PROVIDERS | X | X | X | X | X |  |  |
| Truppo, 2003 | INTERVENE WITH PATIENTS/CONSUMERS TO ENHANCE UPTAKE AND ADHERENCE | X | X | X | X | X |  |  |
| Truppo, 2003 | OBTAIN AND USE PATIENT/CONSUMERS AND  FAMILY FEEDBACK | X | X | X | X | X | X |  |
| Straka, 2005 | CREATE NEW CLINICAL TEAMS | X | X | X | X | X | X |  |
| Straka, 2005 | FACILITATE RELAY OF CLINICAL DATA TO PROVIDERS | X | X | X | X | X |  |  |
| Straka, 2005 | INTERVENE WITH PATIENTS/CONSUMERS TO ENHANCE UPTAKE AND ADHERENCE | X | X | X | X | X |  |  |
| Stockl, 2008 | DISTRIBUTE EDUCATIONAL MATERIALS | X | X | X | X | X |  |  |
| Stockl, 2008 | FACILITATE RELAY OF CLINICAL DATA TO PROVIDERS | X | X | X | X | X | X |  |
| Stephenson, 2009 | DISTRIBUTE EDUCATIONAL MATERIALS | X | X | X |  |  | X |  |
| Stephenson, 2009 | INTERVENE WITH PATIENTS/CONSUMERS TO ENHANCE UPTAKE AND ADHERENCE | X | X | X |  |  |  |  |
| Stacy, 2009 | DISTRIBUTE EDUCATIONAL MATERIALS | X | X | X | X | X |  | X |
| Stacy, 2009 | INTERVENE WITH PATIENTS/CONSUMERS TO ENHANCE UPTAKE AND ADHERENCE | X | X | X | X | X |  | X |
| Shibley, 1997 | CONDUCT EDUCATIONAL OUTREACH VISITS | X | X | X | X | X | X |  |
| Shibley, 1997 | CREATE NEW CLINICAL TEAMS | X | X | X | X |  |  |  |
| Shibley, 1997 | DISTRIBUTE EDUCATIONAL MATERIALS | X | X | X | X | X | X |  |
| Shibley, 1997 | FACILITATE RELAY OF CLINICAL DATA TO PROVIDERS | X | X | X | X | X |  |  |
| Schwed, 1999 | INTERVENE WITH PATIENTS/CONSUMERS TO ENHANCE UPTAKE AND ADHERENCE | X | X | X | X | X |  |  |
| Schectman, 1996 | CONDUCT EDUCATIONAL OUTREACH VISITS | X | X | X | X |  |  |  |
| Schectman, 1996 | CREATE NEW CLINICAL TEAMS | X | X | X | X |  |  |  |
| Schectman, 1996 | INTERVENE WITH PATIENTS/CONSUMERS TO ENHANCE UPTAKE AND ADHERENCE | X | X | X | X | X |  |  |
| Riesen, 2008 | DISTRIBUTE EDUCATIONAL MATERIALS | X | X | X |  |  |  |  |
| Riesen, 2008 | INTERVENE WITH PATIENTS/CONSUMERS TO ENHANCE UPTAKE AND ADHERENCE | X | X | X | X |  |  |  |
| Rachmani, 2005 | DISTRIBUTE EDUCATIONAL MATERIALS | X | X | X | X | X |  |  |
| Rachmani, 2005 | INTERVENE WITH PATIENTS/CONSUMERS TO ENHANCE UPTAKE AND ADHERENCE | X | X | X | X | X |  |  |
| Rachmani, 2005 | REMIND CLINICIANS | X | X | X | X | X |  |  |
| Nordmann, 2000 | DISTRIBUTE EDUCATIONAL MATERIALS | X | X | X | X | X |  |  |
| Nordmann, 2000 | FACILITATE RELAY OF CLINICAL DATA TO PROVIDERS | X | X | X | X | X |  |  |
| Nguyen, 2000 | CONDUCT EDUCATIONAL OUTREACH VISITS | X | X | X |  |  |  |  |
| Nguyen, 2000 | DEVELOP EDUCATIONAL MATERIALS | X | X | X |  |  |  |  |
| Nguyen, 2000 | DISTRIBUTE EDUCATIONAL MATERIALS | X | X | X |  |  |  |  |
| Lindholm, 1996 | CONDUCT EDUCATIONAL OUTREACH VISITS | X | X | X | X | X |  |  |
| Lindholm, 1996 | DISTRIBUTE EDUCATIONAL MATERIALS | X | X | X | X |  |  |  |
| Lima, 2009 | PREPARE PATIENTS/CONSUMERS TO BE ACTIVE PARTICIPANTS | X | X | X | X | X |  |  |
| Lester, 2006 | CHANGE RECORD SYSTEMS | X | X | X | X | X |  |  |
| Lester, 2006 | FACILITATE RELAY OF CLINICAL DATA TO PROVIDERS | X | X | X | X | X |  |  |
| Lester, 2006 | REMIND CLINICIANS | X | X | X | X | X |  |  |
| Kooy, 2013 | INTERVENE WITH PATIENTS/CONSUMERS TO ENHANCE UPTAKE AND ADHERENCE | X | X | X | X | X |  |  |
| Kardas, 2013 | DISTRIBUTE EDUCATIONAL MATERIALS | X | X | X | X | X |  |  |
| Kardas, 2013 | INTERVENE WITH PATIENTS/CONSUMERS TO ENHANCE UPTAKE AND ADHERENCE | X | X | X | X | X |  |  |
| Jakobsson, 2016 | DISTRIBUTE EDUCATIONAL MATERIALS | X | X | X | X | X |  |  |
| Hilleman, 2004 | REMIND CLINICIANS | X | X | X | X | X |  |  |
| Hilleman, 2004 | REVISE PROFESSIONAL ROLES | X | X | X | X | X |  |  |
| Harrison, 2016 | DEVELOP AND ORGANIZE QUALITY MONITORING SYSTEMS | X | X |  | X | X |  |  |
| Harrison, 2016 | INTERVENE WITH PATIENTS/CONSUMERS TO ENHANCE UPTAKE AND ADHERENCE | X | X | X | X | X |  |  |
| Harats, 2005 | CONDUCT EDUCATIONAL MEETINGS | X | X | X | X | X |  |  |
| Goldberg, 2007 | CONDUCT EDUCATIONAL MEETINGS | X | X | X | X | X |  |  |
| Goldberg, 2007 | CONDUCT EDUCATIONAL OUTREACH VISITS | X | X | X | X | X |  |  |
| Goldberg, 2007 | DEVELOP EDUCATIONAL MATERIALS | X | X | X |  |  |  |  |
| Goldberg, 2007 | DISTRIBUTE EDUCATIONAL MATERIALS | X | X | X | X | X |  |  |
| Gitt, 2011 | FACILITATE RELAY OF CLINICAL DATA TO PROVIDERS | X | X | X | X | X |  |  |
| Geber, 2002 | REMIND CLINICIANS | X | X | X | X |  |  |  |
| Gavish, 2002 | INTERVENE WITH PATIENTS/CONSUMERS TO ENHANCE UPTAKE AND ADHERENCE | X | X | X | X | X |  |  |
| Ford, 2001 | FACILITATE RELAY OF CLINICAL DATA TO PROVIDERS | X | X | X |  |  | X |  |
| Etxeberria, 2018 | CONDUCT EDUCATIONAL MEETINGS | X | X | X | X | X |  |  |
| Etxeberria, 2018 | CONDUCT EDUCATIONAL OUTREACH VISITS | X | X | X | X |  |  |  |
| Etxeberria, 2018 | DISTRIBUTE EDUCATIONAL MATERIALS | X | X | X | X |  |  |  |
| Mols, 2015 | INTERVENE WITH PATIENTS/CONSUMERS TO ENHANCE UPTAKE AND ADHERENCE | X | X | X | X | X |  |  |
| Dresser, 2013 | CONDUCT EDUCATIONAL OUTREACH VISITS | X | X | X | X | X |  |  |
| Dresser, 2013 | DEVELOP EDUCATIONAL MATERIALS | X | X | X |  |  |  |  |
| Dresser, 2013 | DISTRIBUTE EDUCATIONAL MATERIALS | X | X | X |  |  |  |  |
| Dresser, 2013 | PROVIDE ONGOING CONSULTATION | X | X | X | X | X |  |  |
| de Velasco, 2004 | CONDUCT EDUCATIONAL MEETINGS | X | X | X | X |  |  |  |
| de Velasco, 2004 | DEVELOP EDUCATIONAL MATERIALS | X | X | X |  |  |  |  |
| de Velasco, 2004 | DISTRIBUTE EDUCATIONAL MATERIALS | X | X | X | X |  |  |  |
| de Velasco, 2004 | INVOLVE PATIENT/CONSUMERS AND FAMILY MEMBERS | X | X | X | X |  |  |  |
| de Lusignan, 2006 | AUDIT AND PROVIDE FEEDBACK | X | X | X |  |  |  |  |
| de Lusignan, 2006 | DEVELOP AND IMPLEMENT TOOLS FOR QUALITY MONITORING | X | X |  |  |  |  |  |
| de Lusignan, 2006 | STAGE IMPLEMENTATION SCALE UP | X | X |  |  |  |  |  |
| Chung, 2011 | CREATE NEW CLINICAL TEAMS | X | X | X | X | X | X |  |
| Chung, 2011 | DISTRIBUTE EDUCATIONAL MATERIALS | X | X | X | X | X |  |  |
| Chung, 2011 | INTERVENE WITH PATIENTS/CONSUMERS TO ENHANCE UPTAKE AND ADHERENCE | X | X | X | X | X |  |  |
| Casebeer, 2009 | DISTRIBUTE EDUCATIONAL MATERIALS (1) | X | X | X |  |  |  |  |
| Casebeer, 2009 | DISTRIBUTE EDUCATIONAL MATERIALS (2) | X | X | X | X | X |  |  |
| Brath, 2013 | CHANGE RECORD SYSTEMS | X | X | X |  | X |  |  |
| Brath, 2013 | INTERVENE WITH PATIENTS/CONSUMERS TO ENHANCE UPTAKE AND ADHERENCE | X | X | X | X | X |  |  |
| Bosworth, 2017 | INTERVENE WITH PATIENTS/CONSUMERS TO ENHANCE UPTAKE AND ADHERENCE | X | X | X | X | X |  |  |
| Bhattacharyya, 2010 | AUDIT AND PROVIDE FEEDBACK | X | X | X |  |  | X |  |
| Bhattacharyya, 2010 | CONDUCT EDUCATIONAL OUTREACH VISITS | X | X | X | X | X |  |  |
| Bassa, 2005 | CHANGE RECORD SYSTEMS |  | X | X | X |  |  |  |
| Bassa, 2005 | DEVELOP AND IMPLEMENT TOOLS FOR QUALITY MONITORING |  | X | X | X |  |  |  |
| Bassa, 2005 | DEVELOP AND ORGANIZE QUALITY MONITORING SYSTEMS | X | X |  |  |  |  |  |
| Bassa, 2005 | FACILITATE RELAY OF CLINICAL DATA TO PROVIDERS | X | X | X | X | X |  |  |
| Bassa, 2005 | OBTAIN FORMAL COMMITMENTS | X | X | X | X |  |  |  |
| Bassa, 2005 | REMIND CLINICIANS | X | X | X | X | X |  |  |
| Andrews, 2017 | CONDUCT EDUCATIONAL OUTREACH VISITS | X | X | X |  |  |  |  |
| Andrews, 2017 | DEVELOP AND ORGANIZE QUALITY MONITORING SYSTEMS | X | X | X | X |  | X |  |
| Andrews, 2017 | INTERVENE WITH PATIENTS/CONSUMERS TO ENHANCE UPTAKE AND ADHERENCE | X | X | X | X | X |  |  |
| Bogden, 1997 | CREATE NEW CLINICAL TEAMS | X | X | X | X |  |  |  |
| Bogden, 1997 | PREPARE PATIENTS/CONSUMERS TO BE ACTIVE PARTICIPANTS | X | X | X | X |  |  |  |
| Bogden, 1997 | REVISE PROFESSIONAL ROLES | X | X | X |  |  |  |  |
| Rehring, 2006 | CREATE NEW CLINICAL TEAMS | X | X | X |  |  |  |  |
| Rehring, 2006 | DEVELOP AND ORGANIZE QUALITY MONITORING SYSTEMS | X | X | X | X | X | X |  |
| Rehring, 2006 | FACILITATE RELAY OF CLINICAL DATA TO PROVIDERS | X | X | X |  | X |  |  |
| Rehring, 2006 | STAGE IMPLEMENTATION SCALE UP | X | X | X |  |  |  |  |
| Hatfield, 2008 | CONDUCT EDUCATIONAL OUTREACH VISITS |  | X | X |  |  |  |  |
| Hatfield, 2008 | CREATE NEW CLINICAL TEAMS | X | X | X | X | X |  |  |
| Aziz, 2012 | AUDIT AND PROVIDE FEEDBACK | X | X | X |  | X | X |  |
| Aziz, 2012 | CREATE NEW CLINICAL TEAMS | X | X | X |  |  |  |  |
| Aziz, 2012 | DEVELOP AND IMPLEMENT TOOLS FOR QUALITY MONITORING | X | X | X |  | X | X |  |
| Aziz, 2012 | DEVELOP AND ORGANIZE QUALITY MONITORING SYSTEMS | X | X |  |  |  |  |  |
| Aziz, 2012 | DEVELOP EDUCATIONAL MATERIALS | X | X | X |  |  |  |  |
| Aziz, 2012 | DISTRIBUTE EDUCATIONAL MATERIALS | X | X | X |  |  |  |  |
| Aziz, 2012 | IDENTIFY AND PREPARE CHAMPIONS | X | X | X |  |  |  |  |
| Aziz, 2012 | MANDATE CHANGE | X | X | X |  |  |  |  |
| Aziz, 2012 | ORGANIZE CLINICIAN IMPLEMENTATION TEAM MEETINGS | X | X | X |  |  |  |  |
| Aziz, 2012 | PROMOTE ADAPTABILITY | X | X |  |  |  |  |  |
| Aziz, 2012 | PURPOSELY REEXAMINE THE IMPLEMENTATION | X | X | X |  |  | X |  |
| Aziz, 2012 | REMIND CLINICIANS | X | X | X |  |  | X |  |
| Aziz, 2012 | USE DATA EXPERTS | X | X | X |  |  |  |  |
| Lappé, 2004 | AUDIT AND PROVIDE FEEDBACK | X | X | X | X |  | X |  |
| Lappé, 2004 | CHANGE RECORD SYSTEMS | X | X | X | X |  |  |  |
| Lappé, 2004 | CONDUCT EDUCATIONAL MEETINGS | X | X | X |  |  |  |  |
| Lappé, 2004 | DEVELOP AND IMPLEMENT TOOLS FOR QUALITY MONITORING | X | X | X | X |  |  |  |
| Lappé, 2004 | DEVELOP AND ORGANIZE QUALITY MONITORING SYSTEMS | X | X | X |  |  |  |  |
| Lappé, 2004 | DEVELOP EDUCATIONAL MATERIALS | X | X | X |  |  |  |  |
| Lappé, 2004 | DISTRIBUTE EDUCATIONAL MATERIALS | X | X | X |  |  |  |  |
| Lappé, 2004 | REMIND CLINICIANS | X | X | X | X |  |  |  |
| Hilleman, 2002 | REMIND CLINICIANS | X | X | X | X | X |  |  |
| FaDeulkner, 2000 | INTERVENE WITH PATIENTS/CONSUMERS TO ENHANCE UPTAKE AND ADHERENCE | X | X | X | X | X |  |  |
| Goswami, 2013 | ALTER PATIENT/CONSUMER FEES | X | X | X |  |  |  |  |
| Goswami, 2013 | DISTRIBUTE EDUCATIONAL MATERIALS | X | X | X | X | X |  |  |
| Goswami, 2013 | INTERVENE WITH PATIENTS/CONSUMERS TO ENHANCE UPTAKE AND ADHERENCE | X | X | X | X | X |  |  |
| Clark, 2014 | ALTER PATIENT/CONSUMER FEES | X | X | X | X | X |  |  |
| Farley, 2012 | ALTER PATIENT/CONSUMER FEES | X | X | X |  | X |  |  |
| Chen, 2010 | CHANGE RECORD SYSTEMS |  | X |  |  |  |  |  |
| Chen, 2010 | FACILITATE RELAY OF CLINICAL DATA TO PROVIDERS | X | X | X |  | X |  |  |
| Chen, 2010 | REMIND CLINICIANS | X | X | X |  | X |  |  |
| Asch, 2015 | ALTER INCENTIVE/ALLOWANCE STRUCTURES | X | X | X |  | X |  |  |
| Asch, 2015 | DEVELOP AND IMPLEMENT TOOLS FOR QUALITY MONITORING | X | X | X |  | X |  |  |
| Asch, 2015 | INTERVENE WITH PATIENTS/CONSUMERS TO ENHANCE UPTAKE AND ADHERENCE | X | X | X |  | X |  |  |
| Choudhry, 2018 | CHANGE RECORD SYSTEMS | X | X | X |  |  |  |  |
| Choudhry, 2018 | CONDUCT EDUCATIONAL MEETINGS | X | X | X |  | X |  |  |
| Choudhry, 2018 | DEVELOP AND ORGANIZE QUALITY MONITORING SYSTEMS | X | X | X |  | X |  |  |
| Choudhry, 2018 | DEVELOP EDUCATIONAL MATERIALS | X | X | X |  | X |  |  |
| Choudhry, 2018 | DISTRIBUTE EDUCATIONAL MATERIALS | X | X | X |  | X |  |  |
| Choudhry, 2018 | FACILITATE RELAY OF CLINICAL DATA TO PROVIDERS | X | X | X |  | X |  |  |
| Choudhry, 2018 | INTERVENE WITH PATIENTS/CONSUMERS TO ENHANCE UPTAKE AND ADHERENCE | X | X | X |  |  |  | X |
| Choudhry, 2018 | REMIND CLINICIANS | X | X | X |  |  |  |  |
| Khanal, 2007 | CREATE NEW CLINICAL TEAMS | X | X | X |  |  |  |  |
| Khanal, 2007 | REVISE PROFESSIONAL ROLES | X | X | X |  |  |  |  |
| Lee, 2006 | CREATE NEW CLINICAL TEAMS | X | X | X | X | X |  |  |
| Lee, 2006 | DISTRIBUTE EDUCATIONAL MATERIALS | X | X | X | X | X |  |  |
| Lee, 2006 | INTERVENE WITH PATIENTS/CONSUMERS TO ENHANCE UPTAKE AND ADHERENCE | X | X | X | X | X |  |  |
| Damush, 2016 | DISTRIBUTE EDUCATIONAL MATERIALS | X | X | X |  |  | X | X |
| Damush, 2016 | INTERVENE WITH PATIENTS/CONSUMERS TO ENHANCE UPTAKE AND ADHERENCE | X | X | X | X | X | X | X |
| Damush, 2016 | TAILOR STRATEGIES | X | X | X |  |  | X | X |
| Mehrpooya, 2018 | INTERVENE WITH PATIENTS/CONSUMERS TO ENHANCE UPTAKE AND ADHERENCE | X | X | X | X | X |  |  |
| Martinez, 2018 | DISTRIBUTE EDUCATIONAL MATERIALS | X | X | X | X | X |  |  |
| Martinez, 2018 | INTERVENE WITH PATIENTS/CONSUMERS TO ENHANCE UPTAKE AND ADHERENCE | X | X | X | X | X |  |  |
| Patel, 2015 | INTERVENE WITH PATIENTS/CONSUMERS TO ENHANCE UPTAKE AND ADHERENCE | X | X | X |  |  |  |  |
| McAlister, 2014 | CREATE NEW CLINICAL TEAMS | X | X | X | X | X |  |  |
| McAlister, 2014 | DISTRIBUTE EDUCATIONAL MATERIALS | X | X | X |  |  |  |  |
| McAlister, 2014 | FACILITATE RELAY OF CLINICAL DATA TO PROVIDERS | X | X | X | X | X |  |  |
| McAlister, 2014 | INTERVENE WITH PATIENTS/CONSUMERS TO ENHANCE UPTAKE AND ADHERENCE | X | X | X | X | X |  |  |
| Shoulders, 2014 | CONDUCT EDUCATIONAL OUTREACH VISITS | X | X | X | X | X |  |  |
| Shoulders, 2014 | FACILITATE RELAY OF CLINICAL DATA TO PROVIDERS | X | X | X | X |  |  |  |
| Derose, 2013 | DISTRIBUTE EDUCATIONAL MATERIALS | X | X | X | X | X |  |  |
| Derose, 2013 | INTERVENE WITH PATIENTS/CONSUMERS TO ENHANCE UPTAKE AND ADHERENCE | X | X | X | X | X |  |  |
| Nieuwkerk, 2012 | DISTRIBUTE EDUCATIONAL MATERIALS | X | X | X | X | X |  |  |
| Nieuwkerk, 2012 | INTERVENE WITH PATIENTS/CONSUMERS TO ENHANCE UPTAKE AND ADHERENCE | X | X | X | X | X |  |  |
| Schmittdiel, 2011 | INTERVENE WITH PATIENTS/CONSUMERS TO ENHANCE UPTAKE AND ADHERENCE | X | X | X |  |  |  |  |
| Coodley, 2008 | FACILITATE RELAY OF CLINICAL DATA TO PROVIDERS | X | X | X |  |  |  |  |
| Coodley, 2008 | INTERVENE WITH PATIENTS/CONSUMERS TO ENHANCE UPTAKE AND ADHERENCE | X | X | X | X | X | X |  |
| Coodley, 2008 | REMIND CLINICIANS | X | X | X |  |  |  |  |
| Willich, 2009 | INTERVENE WITH PATIENTS/CONSUMERS TO ENHANCE UPTAKE AND ADHERENCE | X | X | X | X |  |  |  |
| Hung, 2008 | ALTER PATIENT/CONSUMER FEES | X | X | X | X | X |  |  |
| Hung, 2008 | REMIND CLINICIANS | X | X | X | X | X |  |  |
| Brady, 2005 | AUDIT AND PROVIDE FEEDBACK | X | X | X | X | X | X |  |
| Brady, 2005 | DEVELOP AND ORGANIZE QUALITY MONITORING SYSTEMS | X | X | X |  |  |  |  |
| Brady, 2005 | DISTRIBUTE EDUCATIONAL MATERIALS | X | X | X |  | X |  |  |
| Brady, 2005 | FACILITATE RELAY OF CLINICAL DATA TO PROVIDERS | X | X | X |  | X |  |  |
| Brady, 2005 | REMIND CLINICIANS | X | X | X |  |  |  |  |
| Robinson, 2000 | CREATE NEW CLINICAL TEAMS | X | X | X |  |  |  |  |
| Robinson, 2000 | DISTRIBUTE EDUCATIONAL MATERIALS | X | X | X | X | X |  |  |
| Robinson, 2000 | INTERVENE WITH PATIENTS/CONSUMERS TO ENHANCE UPTAKE AND ADHERENCE | X | X | X | X | X |  |  |
| Birtcher, 2000 | CONDUCT EDUCATIONAL MEETINGS | X | X | X | X | X |  |  |
| Birtcher, 2000 | CONDUCT LOCAL CONSENSUS DISCUSSIONS | X | X | X | X | X |  |  |
| Birtcher, 2000 | CONDUCT LOCAL NEEDS ASSESSMENT | X | X | X |  |  |  |  |
| Birtcher, 2000 | IDENTIFY AND PREPARE CHAMPIONS | X | X |  | X | X |  |  |
| Birtcher, 2000 | INVOLVE EXECUTIVE BOARDS | X | X | X | X | X |  |  |
| Birtcher, 2000 | REMIND CLINICIANS | X | X | X | X | X |  |  |
| Birtcher, 2000 | REVISE PROFESSIONAL ROLES | X | X | X |  |  |  |  |
| Shaffer, 1995 | CREATE NEW CLINICAL TEAMS | X | X | X | X |  |  |  |
| Shaffer, 1995 | DISTRIBUTE EDUCATIONAL MATERIALS | X | X | X | X |  |  |  |
| Shaffer, 1995 | INTERVENE WITH PATIENTS/CONSUMERS TO ENHANCE UPTAKE AND ADHERENCE | X | X | X | X |  |  |  |
| Choe, 2007 | ALTER PATIENT/CONSUMER FEES | X | X | X | X |  |  |  |
| Choe, 2007 | DISTRIBUTE EDUCATIONAL MATERIALS | X | X | X |  | X |  |  |
| Choe, 2007 | INTERVENE WITH PATIENTS/CONSUMERS TO ENHANCE UPTAKE AND ADHERENCE | X | X | X | X | X | X |  |
| Choe, 2007 | REMIND CLINICIANS | X | X | X |  |  |  |  |
| McLeod, 2005 | CREATE NEW CLINICAL TEAMS | X | X | X |  |  |  |  |
| Rabinowitz, 2005 | CREATE NEW CLINICAL TEAMS | X | X | X |  |  |  |  |
| Rabinowitz, 2005 | DISTRIBUTE EDUCATIONAL MATERIALS | X | X | X | X |  |  |  |
| Rabinowitz, 2005 | FACILITATE RELAY OF CLINICAL DATA TO PROVIDERS | X | X | X | X |  |  |  |
| Rabinowitz, 2005 | REMIND CLINICIANS | X | X | X | X |  |  |  |
| Rabinowitz, 2005 | REVISE PROFESSIONAL ROLES | X | X | X | X |  |  |  |
| Ryan, 2003 | CREATE NEW CLINICAL TEAMS | X | X | X |  |  |  |  |
| Ryan, 2003 | INTERVENE WITH PATIENTS/CONSUMERS TO ENHANCE UPTAKE AND ADHERENCE | X | X | X | X |  |  |  |
| Sebregts, 2003 | DISTRIBUTE EDUCATIONAL MATERIALS | X | X | X | X | X |  |  |
| Sebregts, 2003 | PREPARE PATIENTS/CONSUMERS TO BE ACTIVE PARTICIPANTS | X | X | X | X | X |  |  |
| Lowrie, 2014 | ASSESS READINESS AND IDENTIFY BARRIERS AND FACILITATORS | X | X | X |  |  |  |  |
| Lowrie, 2014 | AUDIT AND PROVIDE FEEDBACK | X | X | X | X | X |  |  |
| Lowrie, 2014 | CONDUCT EDUCATIONAL MEETINGS | X | X | X |  | X |  |  |
| Lowrie, 2014 | CONDUCT EDUCATIONAL OUTREACH VISITS | X | X | X | X | X |  |  |
| Lowrie, 2014 | CONDUCT LOCAL NEEDS ASSESSMENT | X | X | X |  |  |  |  |
| Lowrie, 2014 | DEVELOP AND ORGANIZE QUALITY MONITORING SYSTEMS | X | X | X |  |  |  |  |
| Lowrie, 2014 | INFORM LOCAL OPINION LEADERS | X | X | X | X | X |  |  |
| Lowrie, 2014 | REMIND CLINICIANS | X | X | X | X |  |  |  |
| Lowrie, 2014 | REVISE PROFESSIONAL ROLES | X | X | X |  |  |  |  |
| McAlister, 2009 | INFORM LOCAL OPINION LEADERS | X | X | X |  |  |  |  |
| McAlister, 2009 | REMIND CLINICIANS | X | X | X | X |  |  |  |
| Persell, 2015 | CONDUCT EDUCATIONAL MEETINGS | X | X | X | X | X |  |  |
| Persell, 2015 | DEVELOP EDUCATIONAL MATERIALS | X | X | X | X | X |  |  |
| Persell, 2015 | DISTRIBUTE EDUCATIONAL MATERIALS | X | X | X |  | X |  |  |
| Persell, 2015 | FACILITATE RELAY OF CLINICAL DATA TO PROVIDERS | X | X | X | X | X |  |  |
| Persell, 2015 | INTERVENE WITH PATIENTS/CONSUMERS TO ENHANCE UPTAKE AND ADHERENCE | X | X | X |  | X | X |  |
| Persell, 2015 | REVISE PROFESSIONAL ROLES | X | X | X |  |  |  |  |
| Persell, 2015 | WORK WITH EDUCATIONAL INSTITUTIONS | X | X | X |  | X |  |  |
| Villeneuve, 2010 | BUILD A COALITION | X | X | X | X | X |  |  |
| Villeneuve, 2010 | CONDUCT EDUCATIONAL MEETINGS | X | X |  |  | X |  |  |
| Villeneuve, 2010 | CREATE NEW CLINICAL TEAMS | X | X | X | X |  |  |  |
| Villeneuve, 2010 | FACILITATE RELAY OF CLINICAL DATA TO PROVIDERS | X | X | X | X |  |  |  |
| Villeneuve, 2010 | INTERVENE WITH PATIENTS/CONSUMERS TO ENHANCE UPTAKE AND ADHERENCE | X | X | X | X |  |  |  |
| Villeneuve, 2010 | PREPARE PATIENTS/CONSUMERS TO BE ACTIVE PARTICIPANTS | X | X | X | X |  |  |  |
| Osborn, 2018 | CONDUCT EDUCATIONAL MEETINGS | X | X | X |  | X |  | X |
| Osborn, 2018 | CONDUCT LOCAL CONSENSUS DISCUSSIONS | X | X | X |  |  |  | X |
| Osborn, 2018 | DEVELOP EDUCATIONAL MATERIALS | X | X |  |  |  |  | X |
| Osborn, 2018 | DISTRIBUTE EDUCATIONAL MATERIALS | X | X | X |  | X |  | X |
| Osborn, 2018 | INTERVENE WITH PATIENTS/CONSUMERS TO ENHANCE UPTAKE AND ADHERENCE | X | X | X |  |  |  | X |
| Osborn, 2018 | INVOLVE PATIENT/CONSUMERS AND FAMILY MEMBERS | X | X | X |  |  |  | X |
| Osborn, 2018 | OBTAIN AND USE PATIENT/CONSUMERS AND  FAMILY FEEDBACK | X | X | X |  |  |  | X |
| Osborn, 2018 | PROMOTE ADAPTABILITY | X | X |  |  |  |  | X |
| Osborn, 2018 | STAGE IMPLEMENTATION SCALE UP | X | X |  |  |  |  | X |
| Osborn, 2018 | USE ADVISORY BOARDS AND WORKGROUPS | X | X | X |  |  |  | X |
| Paulos, 2005 | CHANGE PHYSICAL STRUCTURE AND EQUIPMENT | X | X |  |  |  |  |  |
| Paulos, 2005 | CONDUCT EDUCATIONAL MEETINGS | X | X | X |  |  |  |  |
| Paulos, 2005 | DEVELOP EDUCATIONAL MATERIALS | X | X | X |  |  |  |  |
| Paulos, 2005 | DISTRIBUTE EDUCATIONAL MATERIALS | X | X | X | X |  |  |  |
| Paulos, 2005 | INTERVENE WITH PATIENTS/CONSUMERS TO ENHANCE UPTAKE AND ADHERENCE | X | X | X | X |  |  |  |

Appendix 6. Risk of bias

| **Article** | **Randomization** | **Deviations from intended effect** | **Missing Outcome Data** | **Measurement of Outcome** | **Selective Reporting** | **Overall** |
| --- | --- | --- | --- | --- | --- | --- |
| Zamora, 2013 | LOW RISK | LOW RISK | LOW RISK | LOW RISK | LOW RISK | LOW RISK |
| Webster, 2010 | LOW RISK | LOW RISK | LOW RISK | LOW RISK | LOW RISK | LOW RISK |
| Stacy, 2009 | LOW RISK | LOW RISK | LOW RISK | LOW RISK | LOW RISK | LOW RISK |
| Schectman, 1996 | LOW RISK | LOW RISK | LOW RISK | LOW RISK | LOW RISK | LOW RISK |
| Riesen, 2008 | LOW RISK | LOW RISK | LOW RISK | LOW RISK | LOW RISK | LOW RISK |
| Rachmani, 2005 | LOW RISK | LOW RISK | LOW RISK | LOW RISK | LOW RISK | LOW RISK |
| Nordmann, 2000 | LOW RISK | LOW RISK | LOW RISK | LOW RISK | LOW RISK | LOW RISK |
| Nguyen, 2000 | SOME CONCERNS | LOW RISK | LOW RISK | LOW RISK | LOW RISK | SOME CONERNS |
| Lester, 2005 | LOW RISK | LOW RISK | LOW RISK | LOW RISK | LOW RISK | LOW RISK |
| Kooy, 2013 | LOW RISK | LOW RISK | LOW RISK | LOW RISK | LOW RISK | LOW RISK |
| Kardas, 2013 | SOME CONCERNS | LOW RISK | LOW RISK | LOW RISK | LOW RISK | SOME CONERNS |
| Jakobsson, 2016 | SOME CONCERNS | SOME CONCERNS | LOW RISK | LOW RISK | LOW RISK | SOME CONERNS |
| Mols, 2015 | SOME CONCERNS | LOW RISK | LOW RISK | LOW RISK | LOW RISK | SOME CONERNS |
| Bogden, 1997 | SOME CONCERNS | LOW RISK | LOW RISK | LOW RISK | LOW RISK | SOME CONERNS |
| Faulkner, 2000 | LOW RISK | LOW RISK | LOW RISK | LOW RISK | LOW RISK | LOW RISK |
| Goswami, 2013 | LOW RISK | LOW RISK | LOW RISK | LOW RISK | LOW RISK | LOW RISK |
| Asch, 2015 | LOW RISK | LOW RISK | LOW RISK | LOW RISK | LOW RISK | LOW RISK |
| Choudhry, 2018 | LOW RISK | LOW RISK | LOW RISK | LOW RISK | LOW RISK | LOW RISK |
| Khanal, 2007 | SOME CONCERNS | LOW RISK | LOW RISK | LOW RISK | LOW RISK | SOME CONERNS |
| Lee, 2006 | LOW RISK | LOW RISK | LOW RISK | LOW RISK | LOW RISK | LOW RISK |
| Damush, 2016 | LOW RISK | LOW RISK | LOW RISK | LOW RISK | LOW RISK | LOW RISK |
| Mehrpooya, 2018 | LOW RISK | LOW RISK | LOW RISK | LOW RISK | LOW RISK | LOW RISK |
| Martinez, 2018 | LOW RISK | LOW RISK | LOW RISK | LOW RISK | LOW RISK | LOW RISK |
| Patel, 2015 | LOW RISK | LOW RISK | LOW RISK | LOW RISK | LOW RISK | LOW RISK |
| McAlister, 2014 | LOW RISK | LOW RISK | LOW RISK | LOW RISK | LOW RISK | LOW RISK |
| Nieuwkerk, 2012 | LOW RISK | LOW RISK | LOW RISK | LOW RISK | LOW RISK | LOW RISK |
| Willich, 2009 | LOW RISK | LOW RISK | LOW RISK | LOW RISK | LOW RISK | LOW RISK |
| Lowrie, 2014 | LOW RISK | LOW RISK | LOW RISK | LOW RISK | LOW RISK | LOW RISK |
| McAlister, 2009 | LOW RISK | LOW RISK | LOW RISK | LOW RISK | LOW RISK | LOW RISK |
| Villeneuve, 2010 | LOW RISK | LOW RISK | LOW RISK | LOW RISK | LOW RISK | LOW RISK |
| Osborn, 2018 | LOW RISK | LOW RISK | LOW RISK | LOW RISK | LOW RISK | LOW RISK |
